# Supplementary material for: Key learnings from an outcome and embedded process evaluation of a direct to beneficiary mobile health intervention among marginalised women in rural Bihar, India
Source: BMJ Open. 2022 Oct 7;12(10):e052336. doi: 10.1136/bmjopen-2021-052336 (PMC9558784; doi:10.1136/bmjopen-2021-052336)
Supplement: Author's manuscript [file bmjopen-2021-052336.draft_revisions.pdf]

BMJ Open

BMJ Open is committed to open peer review. As part of this commitment we make the peer review history of every article we publish publicly available.

When an article is published we post the peer reviewers' comments and the authors' responses online. We also post the versions of the paper that were used during peer review. These are the versions that the peer review comments apply to.

The versions of the paper that follow are the versions that were submitted during the peer review process. They are not the versions of record or the final published versions. They should not be cited or distributed as the published version of this manuscript.

BMJ Open is an open access journal and the full, final, typeset and author-corrected version of record of the manuscript is available on our site with no access controls, subscription charges or pay-per-view fees (http://bmjopen.bmj.com).

If you have any questions on BMJ Open's open peer review process please email info.bmjopen@bmj.com

BMJ Open

Key learnings from an outcome and embedded process evaluation of an interactive voice response intervention among marginalized women in rural Bihar, India

Journal:	*BMJ Open*
Manuscript ID	bmjopen-2021-052336
Article Type:	Original research
Date Submitted by the Author:	28-May-2021

Complete List of Authors:	Irani, Laili; Population Council<br>., Supriya; Population Council<br>Mathur, Ruchika; Population Council<br>Verma, Raj Kumar; Population Council<br>Mohan, Diwakar; Johns Hopkins University Bloomberg School of Public Health<br>Dhar, Diva; Bill & Melinda Gates Foundation<br>Seth, Aaditeshwar; Gram Vaani<br>Chaudhuri , Indrajit; Project Concern International<br>Chaudhury, Mahua; JEEViKA, Bihar Rural Livelihoods Promotion Society, Department of Rural Development, Government of Bihar<br>Purthy, Apolo; JEEViKA, Bihar Rural Livelihoods Promotion Society, Department of Rural Development, Government of Bihar<br>Nanda, Ankit; Population Council<br>Singh, Shivani; Population Council<br>Gupta, Akshay; Gram Vaani<br>LeFevre, Amnesty; International Health

Keywords:	Nutrition < TROPICAL MEDICINE, Human resource management < HEALTH SERVICES ADMINISTRATION & MANAGEMENT, EDUCATION & TRAINING (see Medical Education & Training)

SCHOLARONE™
Manuscripts

I, the Submitting Author has the right to grant and does grant on behalf of all authors of the Work (as defined in the below author licence), an exclusive licence and/or a non-exclusive licence for contributions from authors who are: i) UK Crown employees; ii) where BMJ has agreed a CC-BY licence shall apply, and/or iii) in accordance with the terms applicable for US Federal Government officers or employees acting as part of their official duties; on a worldwide, perpetual, irrevocable, royalty-free basis to BMJ Publishing Group Ltd ("BMJ") its licensees and where the relevant Journal is co-owned by BMJ to the co-owners of the Journal, to publish the Work in this journal and any other BMJ products and to exploit all rights, as set out in our licence.

The Submitting Author accepts and understands that any supply made under these terms is made by BMJ to the Submitting Author unless you are acting as an employee on behalf of your employer or a postgraduate student of an affiliated institution which is paying any applicable article publishing charge ("APC") for Open Access articles. Where the Submitting Author wishes to make the Work available on an Open Access basis (and intends to pay the relevant APC), the terms of reuse of such Open Access shall be governed by a Creative Commons licence – details of these licences and which Creative Commons licence will apply to this Work are set out in our licence referred to above.

Other than as permitted in any relevant BMJ Author's Self Archiving Policies, I confirm this Work has not been accepted for publication elsewhere, is not being considered for publication elsewhere and does not duplicate material already published. I confirm all authors consent to publication of this Work and authorise the granting of this licence.

For peer review only

Key learnings from an outcome and embedded process evaluation of an interactive voice response intervention among marginalized women in rural Bihar, India

Laili Irani, Supriya, Ruchika Mathur, Raj Kumar Verma, Diwakar Mohan, Diva Dhar, Aaditeshwar Seth, Indrajit Chaudhuri, Mahua Roy Chaudhury, Apolo Purthy, Ankit Nanda, Shivani Singh, Akshay Gupta, Amnesty Lefevre

***Primary and corresponding author:**
Laili Irani
Postal address: c/o Population Council, Zone 5A, Ground Floor India Habitat Centre,
Lodi Road, New Delhi, Delhi 110003, India
Email: laili.ir@gmail.com; Telephone number: +91-1124642901, Fax number: +91-1124642903
ORCID iD: https://orcid.org/0000-0002-5472-507X

Co-authors:
Supriya: Population Council, New Delhi, India
Ruchika Mathur: Population Council, New Delhi, India
Raj Kumar Verma: Population Council, New Delhi, India
Diwakar Mohan: Johns Hopkins University, Baltimore, U.S.A.
Diva Dhar: Bill and Melinda Gates Foundation, Seattle, U.S.A.
Aaditeshwar Seth: Gram Vaani, New Delhi, India
Indrajit Chaudhuri: Project Concern International, New Delhi, India
Mahua Roy Chaudhury: JEEViKA, Bihar Rural Livelihoods Promotion Society, Department of Rural Development, Government of Bihar, Patna, India
Apolo Purthy: JEEViKA, Bihar Rural Livelihoods Promotion Society, Department of Rural Development, Government of Bihar, Patna, India
Ankit Nanda: Population Council, New Delhi, India
Shivani Singh: Population Council, New Delhi, India
Akshay Gupta: Gram Vaani, New Delhi, India
Amnesty Lefevre: University of Cape Town Faculty of Science, Cape Town, South Africa

Keywords: mobile phone technology, interactive voice response system, self-help groups, Bihar, behavior change communication

Word count: 7,632 words

For peer review only

ABSTRACT

Introduction

Bihar's poor performance on maternal and child health and nutrition (MCHN) indicators is often attributed to inadequate uptake of correct health practices, caused by poor health seeking behaviors and gaps in knowledge. As mobile phone technology proliferates within rural India, an innovative IVR (Interactive Voice Response) system based mHealth behavior change intervention, Mobile Vaani (MV), was implemented in six blocks of Nalanda district, Bihar between January 2017-November 2018 to raise awareness and catalyze the uptake of key MCHN behaviors in the community by leveraging self-help groups and their federations.

Methods

This paper presents findings from a rigorous outcome evaluation and extensive process evaluation. The process evaluation applied a realist evaluation approach to understand the context in which the intervention was delivered and received and used mixed methods including qualitative in-depth interviews, meeting observations, and IVR data sourced from the technological partners. On the other hand, the outcome evaluation used a quasi-experimental pre-post design with a sample of 4,800 eligible women, aged 15-49 from SHG households, who were married at the time of survey and had a livebirth in the 24 months prior to the survey. The evaluation was powered to measure a 7% or greater net change after 12 months of intervention.

Results

The evaluation found that coverage of the intervention among the target population was low (23%) but when exposed, treatment-on-treated estimates showed positive uptake of 14-19 percent for knowledge-related outcomes. Process evaluation results substantiated these findings and found that the community professionals and program staff facilitating self-help groups played a key role in disseminating

For peer review only - http://bmjopen.bmj.com/site/about/guidelines.xhtml

information about MV and encouraging use among members and users overwhelmingly had a positive attitude toward the program.

Conclusion

The evaluation identified the potential for scaling up the pilot intervention and highlighted key programmatic recommendations, related to technology, training, content, and program design.

For peer review only

For peer review only - http://bmjopen.bmj.com/site/about/guidelines.xhtml

Strengths and limitations of this study

- With the rapid advent of access to mobile phones, sharing of health information using such technology and its integration into self-help groups offer a unique opportunity for reaching marginalized populations through reinforced and targeted messaging on critical health and nutrition behaviors, which continue to lag behind in rural Bihar, India.
- This study uses an external outcome evaluation coupled with a realist process evaluation to determine the impact of an innovative mHealth behavior change intervention implemented in rural Bihar aimed at catalyzing the uptake of key maternal and child health and nutrition behaviors among self-help group members.
- The findings show that as mobile phones can provide 'anytime-anywhere' access to health and nutrition information free of cost, users – primarily self-help group members – are receptive to the program as well as to sharing messages within their communities.
- While the exposure among the intended audience was low, the evaluation utilized advanced analytical approaches to address this challenge as well as capture the process of how the program was integrated among self-help group members within a complex sociocultural environment.

For peer review only

For peer review only - http://bmjopen.bmj.com/site/about/guidelines.xhtml

INTRODUCTION

Emerging evidence suggests that public health programs in rural India that develop and utilize communication strategies have been successful in promoting positive health and nutrition behaviors.[1-4] However, as massive resources are required to reach large audiences with a range of information, programs have often focused on few priority health areas within limited geographies and target groups.[2] To overcome these shortcomings, some programs have employed information communication technology systems such as mobile phones and community radios to reinforce information to target audiences through direct messaging, voice counseling, interactive media, two-way messaging, etc.[1 5-32] Despite these efforts, it has been challenging to reach marginalized populations and many programs have not been evaluated.[33-35] To address these gaps, communication programs are being implemented within women-centric microfinance-based self-help groups (SHGs) with the intention to reach the most marginalized populations in a systematic and sustained manner across a large geography. There are around 6.5 million SHGs in India, with over 70 million members.[36]

Across rural India, SHGs are typically led by a community mobilizer[1] (CM) and often comprise women[2] from marginalized communities, including those in the lowest socioeconomic strata and historically oppressed castes, who live in the same village and converge to save small sums of money periodically.[37] While there are wide variations in the structure, focus and functionality of SHGs, several studies have shown that programs employing communication strategies within SHGs can improve maternal and child health outcomes, including antenatal care visits, early initiation of breastfeeding and clean cord care.[21 38-46] However, some outcomes continue to lag, especially those involving access to supply-side services and change in long-term behaviors, such as family planning and complementary feeding. Additionally, the

[1] Community mobilizers are women who support the functioning of ~10 SHGs within their village. They facilitate weekly meetings for each SHG.
[2] An SHG comprises 8-12 women who live nearby, meet weekly, save small amounts of money to take out loans internally or with banks at small interest rates, and discuss matters related to finance, livelihoods and health.

For peer review only - http://bmjopen.bmj.com/site/about/guidelines.xhtml

amplifying effects that digital health solutions may have on enhancing communication strategies within SHGs are lesser known.

In Bihar a pilot program, Mobile Vaani (MV), was run by a tech firm, Gram Vaani, from January 2017-November 2018 in six blocks of Nalanda district, leveraging an existing health intervention[3] with SHGs that were established and run by government-led entity, JEEViKA. The program augmented SHG monthly meetings through a multipronged approach: (1) equipped CMs with health information content through trainings by program field officers; (2) educated SHG members to receive mobile phone calls with WHO-approved health information content on nutrition[47], family planning and management of diarrhea; (3) and, allowed SHG members to record content onto MV for wider dissemination. Health information calls were made by SHG members to a toll-free number shared by CMs, resulting in a missed call to the system. The system was programed to disconnect the call and initiate a call-back to the user immediately. The MV platform stored the phone numbers, and at periodic intervals pushed out-bound dialed (OBD) calls to the number. Call content spanned across three priority health areas (nutrition, family planning and management of diarrhea) and comprised two types of messages – studio-generated content (SGC)[4] and user-generated content (UGC)[5]. Exposure to such health information sought to reinforce health and nutrition messages women were receiving in meetings, promote wider dissemination (including among other family members and non-SHG members), and foster dialogue among SHG households.

Since its inception, MV has been implemented in the general population across 25 districts in the Indian states of Bihar, Jharkhand and Madhya Pradesh.[48 49] Yet, little is known about its reach, perceptions of content, or its impact on knowledge for key maternal and child health and nutrition (MCHN) practices

[3] Led by Project Concern International, India (PCI) with grant-in-aid support from the Bill & Melinda Gates Foundation (BMGF)
[4] Studio-generated content was recorded by professional artists in a recording studio and was shared in an episodic manner covering a few health and nutrition themes based around the story of a protagonist and their family.
[5] Listeners could react to existing content or record messages of their own, which would undergo moderation and editing following which they would be telecast on MV, referred to as user-generated content.

For peer review only - http://bmjopen.bmj.com/site/about/guidelines.xhtml

among women within SHG households. This paper summarizes findings from an external evaluation of MV conducted from October 2016 to January 2019. We start by presenting evidence on what was implemented, how the intervention was taken up and the contexts within which it was carried out to better identify the underpinning processes which drove observed changes in knowledge.[50-54] We then present findings from an outcome evaluation on the impact of MV on women's knowledge of key MCHN outcomes to determine whether and how a mobile phone intervention could improve knowledge of key practices among women within SHG households.

METHODS

Study setting

Bihar falls below intended targets and national averages for most maternal, newborn and child health indicators. In 2016-17, in rural Bihar, half the children under five and nearly a third (32%) of mothers were underweight as compared to 38% and 27% nationally, respectively.[55 56] One in five (21%) married women reported having an unmet need for family planning – an estimate twice that of the national average. The incidence of diarrhea among children under-5 within the two weeks preceding the survey was 11% and less than half of those cases were managed with oral rehydration solution.[55] As half the state's rural population owns a mobile phone, there is potential to add interventions, such as MV, to SHG meetings.[16 57-59] Given this context and a growing user base, the MV intervention program was designed.

Evaluation design and conceptual framework

Using mixed methods, the evaluation comprised two components (a) process evaluation and (b) outcome evaluation. The process evaluation sought to garner in-depth knowledge on the functioning of MV in implementation districts to aid in the interpretation of outcome results and inform potential replication.[13 50 52-54 60-65] Using a realist evaluation approach, the process evaluation helped in building an overall understanding of (1)the system readiness, understood in terms of CMs adopting MV; (2)behavioral

For peer review only - http://bmjopen.bmj.com/site/about/guidelines.xhtml

response, understood as the communities' response to the program and its diffusion; and (3)contextual factors that act as enablers or barriers (Figure 1).[51 66]

The impact evaluation used a quasi-experimental before-after design to identify changes in knowledge for maternal and child nutrition, family planning, and diarrhea management among SHG members with a child under 2 years of age. Six intervention blocks were selected by the implementers for programming; six comparison blocks were matched by the evaluators based on the socio-demographic characteristics of female literacy, scheduled caste/schedule tribe (SC/ST) and concentration of SHGs by population.

Data collection and sampling

The process evaluation examined the mechanisms of impact through both primary and secondary data analysis. Primary data collected between November 2017-November 2018 included: (1)structured quantitative interviews with CMs, (2)direct observations of SHG meetings facilitated by a trained CM, (3)qualitative IDIs, and secondary data comprised (4)analyses of system generated data on call records from February 2017-July 2018 obtained through the implementors. Table 1 presents sampling details.

Quantitative interviews with CMs were carried out among 116 CMs selected purposively after attending at least two MV trainings. Data were captured in Hindi using laptops by trained female enumerators following written consent.

Direct observations of SHG meetings led by each of the CMs interviewed were observed to determine how information on MV was shared in the meetings. Trained investigators filled in structured, detailed questionnaires to record observations. Verbal consent was sought from all participants.

Qualitative interviews were carried out with various stakeholders (55 CMs, 180 SHG women, 18 program staff), selected purposively. The criteria for selection of CMs was their attendance in at least two MV

For peer review only - http://bmjopen.bmj.com/site/about/guidelines.xhtml

For peer review only

trainings. SHG members who knew of MV were selected for an interview conducted by trained investigators in Hindi. Three key program staff (Block Project Manager, Block Health Nutrition and Sanitation In-charge, MV field officers) across all intervention blocks were interviewed by senior external evaluators. Data were captured through audio recordings following written consent from the respondents. Audio files were transcribed and translated to English. Transcripts were analyzed using Atlas.ti version 6.2.

Secondary analyses were conducted of system-generated interactive voice recording (IVR) data, which included 2.6 million call records from 44,664 unique phone numbers. Three different system-generated datasets were merged and analyzed, namely: (1)call records to and from MV, (2)items heard by users, and (3)content recorded during calls.

For the outcome evaluation, cross-sectional surveys were carried out at two time points: (1) Baseline survey (n=2,436) from October 2017-January 2018; and (2) Endline survey (n=2,498) 12 months following implementation from November 2018-January 2019. Cross-sectional surveys were powered to detect a 7%/more net change in knowledge on core themes among the target population after 12 months of intervention. Assuming 80% power, and a design effect of 1.5, a sample size of 1,200 SHG households in each arm was sufficient to detect this change. Study respondents were eligible women, aged 15-49 years, currently married, had a live birth in the last 24 months preceding the survey and were from SHG households. At endline, women were cross-sectionally selected from the same SHGs.

Structured surveys included modules to assess women's engagement with MV and its impact on their knowledge regarding RMNCH issues such as maternal and child nutrition, family planning and diarrhea management. Data were collected by female enumerators (n=40) with secondary school education/higher and prior quantitative survey experience. Enumerators were grouped in teams of five, including one designated mentor per team, and a supervisor who accompanied them to households, supported logistics,

For peer review only - http://bmjopen.bmj.com/site/about/guidelines.xhtml

and checked completed study questionnaires. All data were captured on mini-laptops using CS-Pro version 7.1 software. Quality control occurred at three levels: (1)supervisory oversight of individual data collectors; (2)10% spot checks of interviews by a team mentor; and (3)outlier analyses of entered data by the core research team.

Variables (Table 1)

Quantitative interviews among CM and observations of SHG meetings

The key outcome of interest was the knowledge of CMs around four key health and nutrition themes MV was targeting. This was captured through 31 knowledge-related questions; with a correct answer to each question garnering a point; the questions were summed up to form a continuous variable. The other outcomes of interest, captured as dichotomous variables, were the ability of CMs to dial a number and to explain MV features during SHG meetings, which were measured from observing SHG meetings led by the same CMs interviewed. The key explanatory variables were number of themes (four--child nutrition, maternal nutrition, family planning and diarrhea management) and number of MV topics (four--purpose of MV, themes covered by MV, how to call and receive messages from MV, and how to record content) on which CMs received trainings. Control variables included CM's age, education, caste, other employment engagements, duration working as CM and partial/full exposure to mobile phones.

Secondary analyses of IVR data

The main aim of this analysis was identifying the lifecycle of a phone number within the system by the number of times it engaged with the program and whether it received call-backs or OBD. In addition, the total number of calls were assessed for indicators like missed calls, call-backs, OBDs, answered calls, duration of listenership and reasons for disconnecting the call.

Cross-sectional surveys

For peer review only - http://bmjopen.bmj.com/site/about/guidelines.xhtml

For the outcome evaluation, the key dependent variables included women's knowledge of the outcomes of interest, i.e. knowledge of: 1)the need to initiate complementary feeding for an infant between 6-8 months, 2)the correct consistency of food for a child 6-12 months old, 3)making a child's food nutrient and energy dense, 4)the need for proper nutrition for pregnant and lactating women, 5) at least two modern spacing methods of family planning, 6)frontline workers (FLW) being sources of short-term family planning methods, e.g. condoms/pills, and 7)symptoms of diarrhea and its management. Key independent variables of interest were the study arm, i.e. intervention or comparison, and the survey rounds, i.e. baseline and endline. Control variables included: women's parity, number of completed years of formal education, any exposure to mass media over the past year, religion, caste, household wealth[6], SHG membership, duration of membership, type of family structure and contact with a frontline worker.[56 67 68]

A sub-analysis of those who recalled listening to MV content at endline was conducted. The variables captured included whether they preferred UGC/SGC, overall satisfaction with MV, timing of listening to MV and topics recalled.

Statistical procedures

Quantitative interviews among CM and observations of SHG meetings

Bivariate and descriptive analyses were conducted on CM quantitative interviews, and SHG meeting observation data, to assess their knowledge on MV themes by their socio-demographic profile, duration of work as CM, their level of training vis-à-vis MV. Additionally, adjusted logistic regressions provided insights on CMs' performance, i.e. their ability to provide trainings on MV during SHG meetings.

[6] The household wealth index was developed through principal component analysis using information on 26 household assets measured for six categories, five housing characteristics and one on asset ownership, taken from a nationally representative tool. Using these data, wealth quintiles were developed based on equal proportions of the population into five categories, from the first quintile being the poorest to the fifth quintile being the richest.

For peer review only - http://bmjopen.bmj.com/site/about/guidelines.xhtml

Secondary analyses of system-generated data

To analyze system-generated data, we first merged large volumes of data on OBD phone calls to and from the system, as well as of content heard and recorded. Cascade analyses of unique mobile numbers was conducted to determine user's engagement with the MV platform over time. For this, a life cycle of each phone number was created. During the journey of each life cycle, all the calls made by MV or made to MV by each unique number were considered as exposures to MV and recorded over time.

Cross-sectional surveys

Differences between women's individual characteristics, i.e. covariates, of the intervention arms between baseline and endline were tested using ordinary least-squares (for continuous variables) and through logistic regression models (for categorical variables). For instance, women's parity and SHG membership duration were measured as continuous variables, and hence tested using ordinary least-squares regression while rest of the variables were categorical, and hence tested using logistic regression model. In addition, the findings were adjusted for random clustering effect at the block and village levels by using multilevel mixed-effects regression analyses.

To assess the net change in the outcome variables from baseline to endline, a treatment on the treated (TOT) approach was applied using instrumental variable (IV) analysis; the IV being random assignment of treatment status, i.e. the study arm thus controlling for endogeneity to program exposure.[69-71] These analyses were adjusted for covariates and accounted for random clustering effect at the block and village levels. All analyses were done using Stata version 16.0.

Ethical approval

This evaluation has been approved by the evaluation agency's institutional review board. The public was not involved in the design, conduct, reporting, or dissemination plans of our research. Prior to an

For peer review only - http://bmjopen.bmj.com/site/about/guidelines.xhtml

interview, participants received written information on the survey as well the assurance of confidentiality. Written consent was taken from all interviewees and interviews were conducted in a private space.

RESULTS

System readiness: How MV was integrated into SHGs

Findings from IDIs suggest that CMs were the key peer educators to integrate MV into SHGs, by sharing various aspects of the intervention with members in SHG meetings. The sociodemographic profile of CMs (Table 2), indicates that CMs were 34 years old on average and half of them were educated above class 10. Around 15% CMs belonged to SC/ST, one-fifth were engaged in secondary occupations and one-third had worked as a CM for 5/more years. Around 82% of CMs received trainings on at least two health and nutrition themes and more than half (55%) received training on 3-4 topics related to MV. Knowledge scores of CMs ranged from 8.5 to 8.7 (out of 31) across all background characteristics. Further, findings indicate that knowledge scores were higher for CMs who received training on more themes and greater number of MV related topics, and had higher education. Multivariate analyses suggested that trainings received by CMs on themes or MV related topics had no significant effect on their ability to provide trainings on dialing a number or explaining MV features during SHG meetings. However, CMs who were educated above class 10 were more likely to provide trainings on ‘dialing a number’ during SHG meetings as compared to those who were educated till class 10 (OR=6.06). Additionally, CMs with 5 years/more experience were more likely to provide trainings on ‘dialing a number (OR=3.8)’ and ‘explaining MV features’ (Table 2).

Findings from CM IDIs also suggest that the number and quality of trainings imparted to CMs resulted in increased knowledge of MV (how to dial in, listen to and record content) as well as correct health and nutrition practices, and greater confidence in engaging with women in the groups (Table 3-A). CMs acknowledged learning new things and feeling more confident as a result of that. Qualitative evidence additionally suggested that the presence of dedicated human resources, i.e. CMs, to promote the

For peer review only - http://bmjopen.bmj.com/site/about/guidelines.xhtml

intervention also ensured that messages were reinforced to the community through (trusted) channels. JEEViKA professionals. in turn, considered sharing information through MV across SHGs an appropriate initiative. They also noted that better coordination in disseminating information on MV, and health and nutrition messages could have greater effect in sharing and retaining messages among SHG women.

Behavioral response: How users engaged with MV

IVR analysis

User engagement was captured by analyzing IVR data which suggests that over few exposures, the proportion of users receiving call-backs decreased, while the proportion of users who received OBD calls increased (Figure 2). An analysis of 2.6 million call records showed that around 19% of the calls were missed calls and 81% calls were made by MV to the users. Of these 81%, figure 3 indicates that a large proportion were OBDs (62%) and the remaining 38% were call-backs. We further explored users' response to OBDs and call-backs and found that even though the system made more OBDs than call-backs, the proportion of users answering the calls was much higher for call-backs as compared to OBD calls, suggesting that call-backs were more cost effective for MV. If calls were initiated by users, 50-60% of the calls were answered as compared to calls that were pushed (20-25%). A quarter of OBD calls were disconnected within 30 seconds of answering, and users spent longer listening to messages on call-backs. Majority of the calls, irrespective of being call-backs or OBDs, were disconnected because of issues at the users' end which included 'normal clearing/no answer/call rejected/user busy'. Additionally, the reasons for call disconnection at MV end were network congestion, incapacity to handle a large volume of calls by primary rate interface lines, and other technical problems within the MV system (Figure 3).

User perceptions at endline

The outcome evaluation also provided insights on women's engagement with MV and found that at endline, 15% (n=173) had ever heard MV content. Their feedback was generally positive and is described

For peer review only - http://bmjopen.bmj.com/site/about/guidelines.xhtml

below through description of their preferences to what and where they preferred listening to MV along with how they heard and shared content with others.

Around 27% women reported that they preferred UGC over SGC, which is also corroborated from qualitative interviews. Women expressed that they enjoyed listening to stories of local women. Around 88% women reported that they liked listening to MV, and cited multiple reasons including it being their only source of news, and a useful medium for information on maternal health, nutrition and sanitation, which they could access at any time free of cost. Further, around 47% women reported that they could listen to MV anytime and from anywhere, and 56% women reported no 'fixed' time for listening to the platform.

As evidenced by the outcome evaluation, women mostly recalled listening to content on the themes of maternal nutrition (38%), child nutrition (35%), family planning (13%) and diarrhea management (12%). Among the users, 77% found health and nutrition messages useful, and 37% women reported listening to MV content along with their family members. Qualitative findings also suggest that women shared information they heard on MV with their family members, neighbors and relatives, and considerable number of women had shared either the MV number or the information heard on MV at least once (table 3-B).

Technological context: Barriers to use

Qualitative findings suggest that there were economic, gendered and comprehension-related barriers, which hampered the uptake of the intervention. The most common reasons women expressed in IDIs for not using MV were lack of time and no apparent benefit. Some women said that they had insufficient balance in their phones to give a missed call and a few women expressed doubts about MV being a free service. Some respondents with migrant husbands found it problematic to be on a MV call as their spouses complained that their (women's) phones were busy when the husbands called. Women also

For peer review only - http://bmjopen.bmj.com/site/about/guidelines.xhtml

expressed apprehension in using mobile phones for long durations in front of male members of the household. Further, IDIs showed that women found it difficult to comprehend SGC; many women complained that they were unable to keep up with the pace of the SGC. Others mentioned that they faced problems when MV messages were shared in Hindi instead of the local language.

Outcome evaluation: Association of program on knowledge of key outcomes

Key characteristics

The findings from outcome evaluation indicate that more than half the women had no formal education, the overwhelming majority belonged to Hindu religion, a third belonged were SC/ST, and the average parity of a woman was three children at baseline across both study arms. The proportion of women belonging to the poorest households was higher in the comparison area at both rounds of survey; at baseline, around 28% of households in the comparison arm belonged to the lowest wealth quintile as compared to 14% in the intervention arm. Mass media exposure of women in the intervention groups were higher than in the comparison group (37% vs 26% at baseline & 47% vs 37% at endline). Furthermore, about two-thirds of the women were living in joint families. Majority of the women had connected with a FLW during their last pregnancy. However, less than half had a postnatal visit with a FLW seven days post-delivery (Table 4). Further, SHG membership was generally high with about 61-78% in each of the study arms; the remaining eligible women interviewed came from a household where another adult female family member was an SHG member. On average, SHG members had been associated with their group for almost three years. All these characteristics were controlled for while carrying out multivariate analyses. The basic socioeconomic characteristics of women captured through the outcome evaluation are comparable in the intervention and comparison arms at baseline. For most of the characteristics, the differences between study arms were not statistically significant suggesting that randomization was successful for the study design (Table 4).

Outcome evaluation results

For peer review only - http://bmjopen.bmj.com/site/about/guidelines.xhtml

The results from outcome evaluation suggest that the intervention was effective in improving women's knowledge on many recommended maternal and child health practices. There was limited exposure (15%) to MV intervention in the intervention arm, presumably due to lack of program readiness, socio-economic conditions, etc. Despite compliance to the actual treatment (i.e. exposure to MV program), random assignment (study arms) strongly predicts actual treatment status; hence ToT approach was used (Table 5). Results suggest that MV intervention was associated with an increase in various knowledge related indicators by 14-19 percentage points as compared to the comparison arm over time. For instance, women's knowledge on the correct consistency of food for a child aged 6-12 months improved by 14 percentage points. Likewise, women's knowledge of at least two modern spacing methods of family planning improved by 18 percentage points.

DISCUSSION

In this paper we have examined the outcomes of the planned intervention as well as the mechanism of program implementation, which sought to increase the knowledge around maternal and child nutrition, family planning, and diarrhea management through mobile phones through SHGs. Using a mixed methods approach, we conducted an embedded process evaluation with an extensive outcome evaluation. The program, despite having been evaluated over a short span of two years, reached a quarter of the intended population and improved their knowledge on many practices around MCHN, indicating that the program was effective to those who were exposed to it. These results are corroborated by other studies showing the effectiveness of health interventions through SHGs as a medium to convey messages to women in marginalized communities.[38 40-43 46]

Changing behaviors within communities takes time, concerted efforts and reiteration of information on correct health practices, which can be achieved through repeated messaging and voice reminders using mobile/mHealth technology through SHGs.[9 72 73] However, it is important to note that mHealth may not function effectively in isolation but instead can be incorporated as a tool for improving indicators within a

For peer review only - http://bmjopen.bmj.com/site/about/guidelines.xhtml

holistic health systems approach. Government and/or private institutions must participate in envisioning and enacting proven mHealth initiatives within national frameworks. This could be achieved by leveraging existing field level cadres such as CMs to reinforce health messages, to reach larger audiences and strengthen health systems, as noted in our study.[74] CMs served as conduits to accelerate and enhance the reach of the program to rural women and were doubly accepted as reliable members of the community. Evidence from the Kilkari program also supports that FLWs may be the primary interlocutors on health issues in villages.[75]

Along with involvement of supportive field cadres, it is also essential to understand the context within which program implementation takes place. The program capitalizes on social capital nurtured within SHGs. Qualitative interviews with women SHG members revealed that women responded relatively well to the program as evidenced by being able to remember and relay content, recording content, and sharing it with friends and neighbors. Components of the program such as entertainment and UGC that catered to and were customized for the intended audience acted as key drivers of the program within the communities. Users responded well to the ease and flexibility of access. However, the program was met with several existing contextual barriers such as lack of mobile literacy, poor phone infrastructure, inability to use mobile phones, gender and cultural barriers, lack of time, among others.[76] Other studies also suggest that women face many difficulties related to mobile phones, which may restrict their access of mHealth technologies or initiatives.[74 75] Women in rural areas having functional mobile phones mostly only use it for receiving calls due to poor phone literacy. Similar contextual factors have been highlighted in explaining low outreach of mass media as well, including newspapers, radio and television, among women in rural Bihar.[2 55 56]

The extensive evaluation also helped highlight the potential for scaling up the intervention more effectively. *First,* a space should be identified where a wide community can be reached that promotes ownership and sustenance of the intended intervention. *Second*, evidence-based and systematic planning

For peer review only - http://bmjopen.bmj.com/site/about/guidelines.xhtml

is needed to design a robust program to achieve its desired take-up by reaching intended audiences through capacity building and extensive promotion of the program. Adequate coverage of the program is essential for success. *Third*, the content being played through the IVR system needs to focus on topics of interest and be relatable to the audience to sustain its programming. As the audience expands to new populations, this will require additional formative research and continued monitoring to tweak the program and allow for mid-course corrections. *Fourth*, for optimal results, the program ought to coordinate its content development and dissemination with any parallel program efforts disseminating health and nutrition information to the intended audience. *Fifth*, the program needs to identify and account for various socio-demographic and economic differences when considering scaling up the program with the intent of reaching different audiences equally and not exacerbating disparities of access across socio-economic characteristics. Other scholars have also captured this phenomenon of intersecting inequalities, especially in terms of gendered phone access gaps and financial constraints.[75 77] *Sixth*, a robust registration and follow-up system of registered phone numbers could be pivotal in ensuring that messages reach their intended audience at the appropriate time. Furthermore, results from the IVR analyses show that listener engagement is higher in call-backs as opposed to OBD. The MoTECH study highlighted that future programs should consider ways to improve the accuracy and completeness in data reporting as well as linkages with existing health information systems which collect data on service utilization and reported practices.[14] Findings from this evaluation echo these recommendations.

Though intervention and comparison arms were matched successfully, with the treatment and control groups generally comparable for their basic characteristics, this evaluation did have some limitations. Baseline and endline samples were not always balanced across the intervention and comparison arms, but key covariates were controlled and accounted for in the analysis. The study used fresh cross-sections of respondents at both time points of survey; however, we ensured that respondents at endline belonged to the same longitudinal SHG samples from baseline, therefore were representative of similar geographic and socio-economic conditions. As the study only engaged SHG members, it may not have considerable

For peer review only - http://bmjopen.bmj.com/site/about/guidelines.xhtml

external validity outside of this population. The exposure among the intended audience was low, but this was adjusted for in the analyses using IV regression. The recall bias among respondents was also accounted for through rigorously trained investigators and pilot testing the survey tools, which were formulated based on established modules from similar surveys.

Conclusion

When introduced to MV through SHGs, women's knowledge of key health and nutrition outcomes improved. The study provides evidence that mobile-based interventions could evolve into an effective modality to encourage correct practices and have potential to be scaled up to reach marginalized populations through systematic planning and extensive efforts. However, it would be advisable for programs to remain conscious of the context, and enablers/barriers driven by socio-economic and gendered realities of the target audience, before operationalizing capital intensive interventions. Concerted efforts introduced among a marginalized population can result in a multiplicative effect to reap exponential benefits in the future.

For peer review only

For peer review only - http://bmjopen.bmj.com/site/about/guidelines.xhtml

FIGURES and TABLES

Figure 1: Conceptual framework outlining key components of process and outcome evaluation

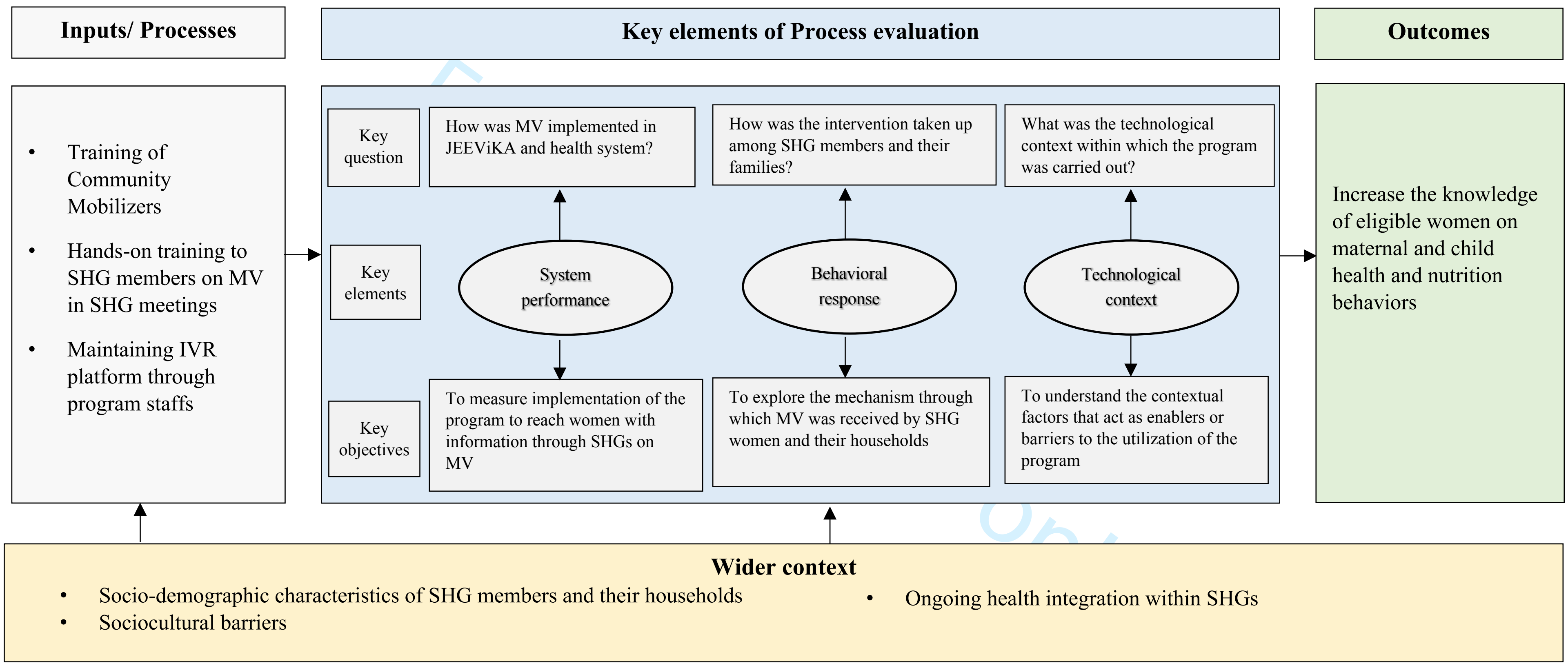

For peer review only - http://bmjopen.bmj.com/site/about/guidelines.xhtml

Table 1: Details of data collection points

Data source	Study population	Sample	Date of data collection	Key outcomes	Key explanatory variables	Control variables
**Process evaluation**						
**Structured quantitative interviews**	Community mobilizers (CM)	116	November 2017 – November 2018	Knowledge score of CM around MV themes (Range: 0-31)	• Number of trainings received by CM on health and nutrition themes (0-1 theme, 2 themes, 3-4 themes)<br>• Number of trainings received by CM on MV topics (0-2 topics, 3-4 topics)	• Age (mean age)<br>• Education (1-10th std, 11th-12th std, graduate and above)<br>• Caste (SC/ST, others)<br>• Engaged in other occupation (yes/no)<br>• Duration of working as CM (0-2 years, 3-4 years, 5 or more years)<br>• Exposure to mobile phone (no/partial exposure, full exposure, i.e. having a personal phone, having access to a phone at all times, and ability to dial a number)
**Direct observations**	SHG meetings	116	November 2017 – November 2018	• CM's ability to dial a number (Yes/No)<br>• CM's ability to explain MV features (Yes/No)		
**In-depth qualitative interviews**	CMs<br>SHG members<br>Program staff	55<br>180<br>18	November 2017 – November 2018			
**System generated interactive voice recording data**	Phone numbers	2.6 million calls<br>44,664 unique phone numbers/ users	February 2017 – July 2018	• Missed calls - user called the system which generated a missed call<br>• Call backs - system called the user following a missed call<br>• out-bound calls - system-generated random calls to registered numbers at periodic intervals	• Answered calls - user answered incoming call<br>• Unanswered calls - user did not answer incoming call<br>• Duration of listenership, in minutes<br>• Reasons for disconnecting the call - network issues, MV issues, user issues	
**Outcome evaluation**						

For peer review only - http://bmjopen.bmj.com/site/about/guidelines.xhtml

**Structured quantitative interviews**	Eligible women	Baseline: 2436 women<br>Endline: 2498 women	Baseline: October 2016 – January 2017<br>Endline: November 2018 – January 2019	Knowledge on:<br>• Complementary feeding (Yes/No)<br>• Correct consistency of food (Yes/No)<br>• Preparing nutrient rich and dense food (Yes/No)<br>• Maternal nutrition (Yes/No)<br>• Modern family planning (Yes/No)<br>• FLW as source of FP methods (Yes/No)<br>• Diarrhea management (Yes/No)	• Study arm (Intervention and comparison arm)<br>• Survey time (baseline and endline)	• Women's parity (Range: 0-9)<br>• Number of completed years of formal education (1-5 years, 6-9 years, ≥10 years)<br>• Any exposure to mass media (i.e. radio, television or newspaper) over the past year (Yes/No)<br>• Religion (Hindu, Others)<br>• Scheduled caste/tribe (Yes/No)<br>• Household wealth (First, second, third, fourth, fifth quintiles)<br>• SHG membership (Yes/No)<br>• Duration of membership (Range: 0 – 180 months)<br>• Nuclear family structure (Yes/No),<br>• Contact with a frontline worker during pregnancy (Yes/No)<br>• Contact with a frontline worker within 7 days of delivery (Yes/No)

For peer review only

Table 2: Findings showing association of characteristics of community mobilizers to sharing MV in SHG meetings (N=116)

Community mobilizers' (CM) background characteristics	Knowledge on MV themes and observations of SHG meetings		Ability of CMs to give training on proposed topics during SHG meetings			
	Average knowledge score Range (0-31)	CMs observed giving MV training in SHG meetings	Dial a number		Explain MV features	
	Mean (SD)	Proportion (%)	Proportion (%)	Odds ratio (95% CI)	Proportion (%)	Odds ratio (95% CI)
**Number of themes on which CMs received trainings, %**						
**0 – 1 theme®**	8.2 (1.11)	12.1	57.1		64.3	
**2 themes**	8.6 (1.22)	69.0	41.3	1.07 (0.24, 4.74)	63.8	2.94 (0.62, 14.03)
**3 – 4 themes**	8.9 (0.87)	19.0	31.8	0.59 (0.1, 3.44)	45.5	1.58 (0.27, 9.37)
**Number of topics related to MV on which CM received trainings, %**						
**0 – 2 topics®**	8.5 (1.21)	37.1	39.5		67.4	
**3 – 4 topics**	8.7 (1.11)	62.9	42.5	1.77 (0.7, 4.45)	56.2	1.08 (0.41, 2.82)
**Age (mean, SD)**	8.6 (1.16)	33.3 (8.45)	31.7 (8.18)	0.95 (0.9, 1.01)	33.0 (7.95)	0.96 (0.91, 1.03)
**Caste, %**						
**SC/ST®**	8.5 (1.21)	12.9	46.7		66.7	
**Others**	8.6 (1.15)	87.1	40.6	0.50 (0.12, 2.00)	59.4	0.39 (0.09, 1.65)
**Education, %**						
**1st – 10th standard®**	8.5 (1.05)	44.0	29.4		54.9	
**11th – 12th standard**	8.7 (1.29)	33.6	46.2	2.18 (0.79, 5.98)	64.1	1.8 (0.63, 5.15)
**College and above**	8.7 (1.15)	22.4	57.7	6.06* (1.64, 22.4)	65.4	3.35 (0.85, 13.26)
**Exposure to mobile, %**						
**No/partial exposure®**	8.7 (1.55)	6.9	37.5		50.0	
**Full exposure**	8.6 (1.11)	93.1	41.7	2.11 (0.29, 15.57)	61.1	1.27 (0.2, 8.01)
**Engaged in other occupation, %**						
**Yes®**	8.5 (1.03)	31.0	50.0		69.4	
**No**	8.6 (1.19)	69.0	37.5	0.23* (0.08, 0.69)	56.3	0.29* (0.09, 0.92)
**Duration of work as a CM (years), %**						
**0-2 years®**	8.5 (1.23)	27.6	28.1		31.3	
**3-4 years**	8.8 (0.96)	41.4	43.8	2.60 (0.87, 7.74)	68.8	7.14* (2.37, 21.5)
**5+ years**	8.5 (1.27)	31.0	50.0	3.81* (1.07, 13.52)	75.0	11.24* (2.91, 43.39)

Note: ® : reference category for logistic regression analysis
*: odds ratios are significant at p ≤0.05

For peer review only

Table 3: Quotes from in-depth interviews

Thematic reference in paper	Type of IDI	Identifier	Quote
3-A. System readiness: How MV was integrated into SHGs			
	**CM**	41 years of age, 10 years of education	*Earlier I didn't have so much knowledge. Didi told us about food, washing our hands and clothes, and that children should be given the first milk of the mother. Now we have gained so much knowledge.*
	**BPM**	N/A	*If motivated correctly, CM can do wonders, as they go door-to-door and spread awareness. … With correct guidelines and structure, we can see [great] results.*
	**Woman**	30 years of age, 12 years of education	*CM is a homemaker. So, she spoke to us in the local dialect. We were all able to understand how we are supposed to dial the number, including the illiterate women of the group [SHG].*
	**BHNSI**	N/A	*The films we are running about nutritious diet of women should play on Mobile Vaani as well because our CM is also discussing about nutritious diet with the women of the groups.*
3-B. Behavioral response: How users engaged with MV			
**User Preference**	**Woman**	38 years of age, 10 years of education	*I mostly like the voice of different women from villages as I feel motivated by listening to their stories. I also feel I should record my own voice just like they do.*
	**Woman**	35 years of age, 9 years of education	*I like the studio voice more. The village women speak in breaks and they don't speak very well. But the studio sister speaks well and explains things very nicely.*
**Content heard and shared**	**Woman**	30 years of age, 3 years of education	*I have also discussed [about what I hear on MV] with my mother-in-law and sisters-in-law. My younger sister-in-law has a small baby and she needs to feed her baby twice or thrice in a day. She should feed her baby properly so the baby will remain healthy.*
3-C. Contextual factors: Barriers to use			
**Barriers to use**	**Woman**	35 years of age, 8 years of education	*You know how husbands are! They will start a fight with us if we are always busy with the mobile. I use it only in his absence and don't even touch it when he is home.*
	**MV FO**	N/A	*I have been working in the field for almost one and a half years here. Many women know about MV, but still there are many who don't even know how to use a mobile. Either they don't have a mobile of their own or they just know how to answer the call and disconnect.*

Note: These quotes are an illustrative sample from a universe of 253 in-depth interviews (IDIs). IDIs were conducted with the following respondents: women, community mobilizers, key informants including Block Project Managers (BPM), Block Health Nutrition and Sanitation In-charge (BHNSI), Field Officers (FO).

For peer review only

Figure 2: Cascade of unique numbers engaging with MV over the course of the program

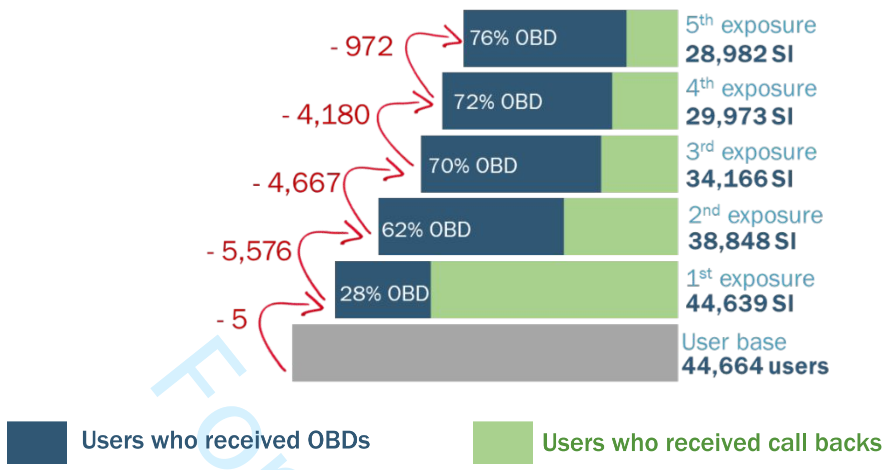

Figure 3: User's engagement with MV system

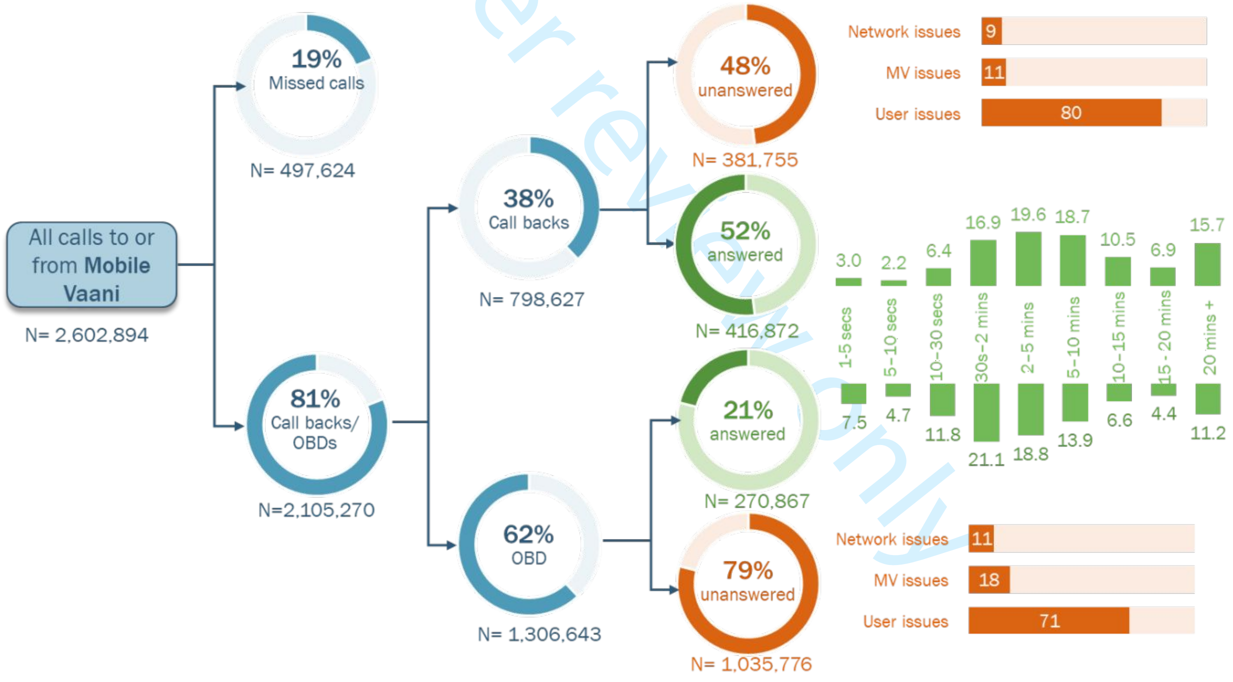

For peer review only

Table 4: Socio-demographic characteristics of the study sample for outcome evaluation

Socio-demographic characteristics	Baseline			Endline		
	Intervention (A) (N=1,249)	Comparison (B) (N=1,187)	p-value A vs B	Intervention (C) (N=1,188)	Comparison (D) (N=1,310)	p-value A vs C
**Women's parity, Mean (S.D.)**	2.95 (1.68)	3.06 (1.74)	0.142	2.81 (1.58)	2.91 (1.65)	0.008
**Women's education, %**						
**No formal education**	56.0	57.5	0.535	47.0	49.4	<0.001
**1-5 years**	12.3	14.5	0.112	13.9	14.7	0.200
**6-9 years**	15.8	15.2	0.729	20.0	20.2	0.005
**>=10 years of schooling**	15.9	12.8	0.064	19.2	16.7	0.020
**Any mass media exposure, %**						
**No**	62.9	74.5	<0.001	53.4	63.0	<0.001
**Yes**	37.1	25.5	<0.001	46.6	37.0	<0.001
**Belongs to Hindu religion, %**	98.5	91.1	<0.001	99.2	90.8	0.136
**Households wealth quintile, %**						
**First**	13.6	28.4	<0.001	15.4	24.7	0.259
**Second**	18.3	21.1	0.126	19.6	20.0	0.383
**Third**	20.3	19.0	0.468	20.5	19.4	0.870
**Fourth**	23.8	15.8	<0.001	23.0	17.3	0.669
**Fifth**	24.1	15.7	<0.001	21.5	18.6	0.122
**Scheduled caste/tribe, %**						
**No**	68.6	69.3	0.815	69.6	65.5	0.654
**Yes**	31.4	30.7	0.815	30.4	34.5	0.654
**Woman herself is an SHG member, %**						
**No**	31.9	21.9	<0.001	39.3	33.5	<0.001
**Yes**	68.1	78.1	<0.001	60.7	66.5	<0.001
**Women's SHG membership duration in months, mean (S.D.)**	34.8 (20.1)	26.9 (17.9)	<0.001	38.5 (22.6)	36.1 (20.5)	0.001
**Nuclear family structure, %**						
**No**	67.4	57.3	<0.001	71.6	65.0	0.007
**Yes**	32.6	42.7	<0.001	28.4	35.0	0.007
**Any contact with FLW during pregnancy, %**						
**No**	27.8	43.0	<0.001	35.7	37.9	<0.001
**Yes**	72.2	57.0	<0.001	64.3	62.1	<0.001
**Any contact with FLW within 7 days of delivery, %**						
**No**	58.1	54.8	0.243	55.8	48.2	0.282
**Yes**	41.9	45.2	0.243	44.2	51.8	0.282

Note: Differences in groups at baseline and endline were tested by using ordinary least-squares regression models (continuous variables) or logit regression models (categorical variables), adjusting for clustering effect at the block and village level; S.D.: standard deviation

For peer review only - http://bmjopen.bmj.com/site/about/guidelines.xhtml

Table 5: Results from treatment-on-the-treated effect of Mobile Vaani intervention on knowledge of selected maternal and newborn health practices for outcome evaluation, 2017-2019

Indicators	Intervention		Comparison			
	Baseline (N=1,249)	Endline (N=1,188)	Baseline (N=1,187)	Endline (N=1,310)	TOT estimate	p-value
Knowledge indicators, %						
**Need to initiate complementary feeding for an infant at 6-8 months**	82.5	79.6	83.5	84.4	-12.1 (-25.2, 0.9)	0.069
**Correct consistency of food is for a child aged 6-12 months**	55.6	56.4	56.0	50.2	14.4 (-6.1, 35)	0.169
**To make a child's food nutrient and energy dense**	72.6	79.8	74.3	72.2	18.8 (0.4, 37.2)	0.045
**Proper nutrition of pregnant and lactating women**	73.6	77.0	69.5	81.0	-0.3 (-16.8, 16.2)	0.971
**At least two modern spacing methods of family planning**	90.3	89.4	84.8	88.6	17.6 (4.7, 30.5)	0.008
**Frontline worker having stocks of condoms and pills**	8.1	14.9	9.2	12.8	2.8 (-9.3, 15)	0.647
**Symptoms of diarrhea and its management**	12.1	17.9	16.5	16.2	-15.2 (-30.9, 0.4)	0.056

Note: Estimates are adjusted for women's parity, education, religion, caste, family structure, wealth quintile, mass media exposure, respondent's membership in groups and duration of SHG membership, FLW contacts during pregnancy and after delivery, accounting for clustering effect at block and village level.
Treatment-on-treated effect are estimated based on instrument variable regression analysis and adjusted for women's parity, education, religion, caste, family structure, wealth quintile, mass media exposure, respondent's membership in groups and duration of SHG membership, FLW contacts during pregnancy and after delivery, accounting for clustering effect at block and village level.

For peer review only

For peer review only - http://bmjopen.bmj.com/site/about/guidelines.xhtml

Contributors: LI served as the PI of the study designing the evaluation, overseeing the data collection, leading the analysis and interpretation of results. LI was the primary author of the paper, had full access to the data, controlled the decision to publish and accepts full responsibility for the conduct of this study, as the guarantor. S, RM, RKM, AN and SS led the data collection, contributed to data analysis and visualization, and wrote early drafts of the methods and results sections of the manuscript. DM contributed to data analysis, interpretation of results and critical revision of the manuscript. DD and AL contributed to the plan and design of the study, contributed to data analysis, interpretation of results and critical revision of the manuscript for important intellectual content. AS, IC, MRC, AP, AG contributed to designing the study, assisted the evaluators to collect primary data and access secondary data, and provided critical insight into the interpretation of the results and revisions of the manuscript. All authors have reviewed and approved the final version of the manuscript.

Funding: The authors were funded by the Bill and Melinda Gates Foundation to carry out this study.

Competing interests: None declared.

Ethics approval: The study was approved and annually reviewed by the evaluation agency's institutional review board to which the primary author belongs.

Patient and public involvement: Patients and/or the public were not involved in the design, or conduct, or reporting, or dissemination plans of this research.

For peer review only

For peer review only - http://bmjopen.bmj.com/site/about/guidelines.xhtml

Patient consent for publication: Not required.

Provenance and peer review: Not commissioned; externally peer reviewed.

Data availability statement: Data are available upon request from the corresponding author. Requests for sharing data must be made of specific grounds, either (1) with the aim of corroborating the study results in the interest of public health, or (2) with the intention to answer new research questions. Sufficient information needs to be provided to confirm that the request is made for one of the above-mentioned purposes, including a sound justification, a protocol on the research for which the data will be used and a plan for quality control checks, as applicable.

For peer review only

For peer review only - http://bmjopen.bmj.com/site/about/guidelines.xhtml

REFERENCES

1. Gurman TA, Rubin SE, Roess AA. Effectiveness of mHealth Behavior Change Communication Interventions in Developing Countries: A Systematic Review of the Literature. *Journal of Health Communication* 2012;17(sup1):82-104. doi: 10.1080/10810730.2011.649160
2. Khan M, Bhatnagar I, Hazra A, et al. Situation analysis of behavior change communication activities in Bihar: A step towards developing a comprehensive BCC strategy. New Delhi: Population Council, 2012.
3. Mozumdar A, Khan ME, Mondal SK, et al. Increasing knowledge of home based maternal and newborn care using self-help groups: Evidence from rural Uttar Pradesh, India. *Sexual & Reproductive Healthcare* 2018;18:1-9. doi: https://doi.org/10.1016/j.srhc.2018.08.003
4. Pillai P. LEADING 21ST CENTURY HEALTHCARE: APP-CENTRIC CARE: "MOBILE HEALTH": The impact of Smartphone applications on health-a futuristic perspective on leading in complex healthcare system. *International Journal of Management Research and Reviews* 2012;2(4):518.
5. Agarwal S, Perry HB, Long L-A, et al. Evidence on feasibility and effective use of mHealth strategies by frontline health workers in developing countries: systematic review. *Tropical Medicine & International Health* 2015;20(8):1003-14. doi: 10.1111/tmi.12525
6. Avula R, Menon P, Saha KK, et al. A Program Impact Pathway Analysis Identifies Critical Steps in the Implementation and Utilization of a Behavior Change Communication Intervention Promoting Infant and Child Feeding Practices in Bangladesh. *The Journal of Nutrition* 2013;143(12):2029-37. doi: 10.3945/jn.113.179085
7. Chambers DA, Glasgow RE, Stange KC. The dynamic sustainability framework: addressing the paradox of sustainment amid ongoing change. *Implementation Science* 2013;8(1):117. doi: 10.1186/1748-5908-8-117
8. Donabedian A. The Quality of Care: How Can It Be Assessed? *JAMA* 1988;260(12):1743-48. doi: 10.1001/jama.1988.03410120089033
9. Feroz A, Perveen S, Aftab W. Role of mHealth applications for improving antenatal and postnatal care in low and middle income countries: a systematic review. *BMC Health Services Research* 2017;17(1):704. doi: 10.1186/s12913-017-2664-7
10. Feroz A, Rizvi N, Sayani S, et al. Feasibility of mHealth intervention to improve uptake of antenatal and postnatal care services in peri-urban areas of Karachi: A qualitative exploratory study. *Journal of Hospital Management and Health Policy* 2017;4(1)
11. Florez-Arango JF, Iyengar MS, Dunn K, et al. Performance factors of mobile rich media job aids for community health workers. *Journal of the American Medical Informatics Association* 2011;18(2):131-37. doi: 10.1136/jamia.2010.010025
12. Källander K, Tibenderana JK, Akpogheneta OJ, et al. Mobile health (mHealth) approaches and lessons for increased performance and retention of community health workers in low-and middle-income countries: a review. *Journal of medical Internet research* 2013;15(1):e17.
13. Labrique AB, Vasudevan L, Kochi E, et al. mHealth innovations as health system strengthening tools: 12 common applications and a visual framework. *Global Health: Science and Practice* 2013;1(2):160. doi: 10.9745/GHSP-D-13-00031
14. LeFevre AE, Mohan D, Hutchful D, et al. Mobile Technology for Community Health in Ghana: what happens when technical functionality threatens the effectiveness of digital health programs? *BMC Medical Informatics and Decision Making* 2017;17(1):27. doi: 10.1186/s12911-017-0421-9
15. Mahanta LB, Choudhury M, Borthakur S, et al. Can communication media improve the KAP status of frontline health workers? A cross-sectional study on MCH indicators from Assam, India. *South East Asia Journal of Public Health* 2015;5(2):57-64.
16. Malhotra A, Sharma R, Srinivasan R, et al. Widening the arc of indigenous communication: Examining potential for use of ICT in strengthening social and behavior change communication efforts with

For peer review only - http://bmjopen.bmj.com/site/about/guidelines.xhtml

marginalized communities in India. *The Electronic Journal of Information Systems in Developing Countries* 2018;84(4):e12032. doi: 10.1002/isd2.12032
17. Mangwi Ayiasi R, Kolsteren P, Batwala V, et al. Effect of Village Health Team Home Visits and Mobile Phone Consultations on Maternal and Newborn Care Practices in Masindi and Kiryandongo, Uganda: A Community-Intervention Trial. *PLoS One* 2016;11(4):e0153051-e51. doi: 10.1371/journal.pone.0153051
18. Maslowsky J, Frost S, Hendrick CE, et al. Effects of postpartum mobile phone-based education on maternal and infant health in Ecuador. *Int J Gynaecol Obstet* 2016;134(1):93-98. doi: 10.1016/j.ijgo.2015.12.008 [published Online First: 2016/04/08]
19. Mildon AM. Use of Mobile Phone Technology to Support Improved Infant and Young Child Feeding Practices in Low-and Middle-income Countries: A Scoping Review. University of Toronto (Canada), 2016.
20. Mitchell M, Hedt-Gauthier BL, Msellemu D, et al. Using electronic technology to improve clinical care – results from a before-after cluster trial to evaluate assessment and classification of sick children according to Integrated Management of Childhood Illness (IMCI) protocol in Tanzania. *BMC Medical Informatics and Decision Making* 2013;13(1):95. doi: 10.1186/1472-6947-13-95
21. More NS, Bapat U, Das S, et al. Community Mobilization in Mumbai Slums to Improve Perinatal Care and Outcomes: A Cluster Randomized Controlled Trial. *PLOS Medicine* 2012;9(7):e1001257. doi: 10.1371/journal.pmed.1001257
22. Mushamiri I, Luo C, Iiams-Hauser C, et al. Evaluation of the impact of a mobile health system on adherence to antenatal and postnatal care and prevention of mother-to-child transmission of HIV programs in Kenya. *BMC Public Health* 2015;15(1):102. doi: 10.1186/s12889-015-1358-5
23. Nair N, Darak S, Parsekar S, et al. Effectiveness of Behaviour Change Communication interventions in improving the delivery of health messages for ante-natal care in limited literacy settings: An evidence summary. London: EPPI-Centre: Social Science Research Unit, UCL Institute of Education, University College London, 2017.
24. Pagliari C, Sloan D, Gregor P, et al. What is eHealth (4): a scoping exercise to map the field. *Journal of medical Internet research* 2005;7(1):e9-e9. doi: 10.2196/jmir.7.1.e9
25. Philbrick WC. MHealth and MNCH: State of the Evidence. Trends, Gaps, Stakeholder Needs, and Opportunities For Future Research on the Use of Mobile Technology to Improve Maternal, Newborn, and Child Health. Washington: UN Foundation., 2013.
26. Rahman A, Leppard M, Rashid S, et al. Community perceptions of behaviour change communication interventions of the maternal neonatal and child health programme in rural Bangladesh: an exploratory study. *BMC Health Services Research* 2016;16(1):389. doi: 10.1186/s12913-016-1632-y
27. Rogers EM, Vaughan PW, Swalehe RMA, et al. Effects of an Entertainment-education Radio Soap Opera on Family Planning Behavior in Tanzania. *Studies in Family Planning* 1999;30(3):193-211. doi: 10.1111/j.1728-4465.1999.00193.x
28. Rothstein JD, Jennings L, Moorthy A, et al. Qualitative Assessment of the Feasibility, Usability, and Acceptability of a Mobile Client Data App for Community-Based Maternal, Neonatal, and Child Care in Rural Ghana. *International Journal of Telemedicine and Applications* 2016;2016:2515420. doi: 10.1155/2016/2515420
29. Singh N, Labrique A, Mehra S, et al. Can an Integrated mHealth System Increase Utilization of Antenatal care services and Promote Safe Delivery? 142nd APHA Annual Meeting and Exposition. New Orleans, LA, 2014.
30. Smith C, Ngo TD, Gold J, et al. Effect of a mobile phone-based intervention on post-abortion contraception: a randomized controlled trial in Cambodia. *Bull World Health Organ* 2015;93(12):842-50A. doi: 10.2471/BLT.15.160267 [published Online First: 2015/10/15]

For peer review only - http://bmjopen.bmj.com/site/about/guidelines.xhtml

For peer review only

31. Uddin MJ, Shamsuzzaman M, Horng L, et al. Use of mobile phones for improving vaccination coverage among children living in rural hard-to-reach areas and urban streets of Bangladesh. *Vaccine* 2016;34(2):276-83. doi: 10.1016/j.vaccine.2015.11.024 [published Online First: 2015/11/29]
32. Vaughan PW, Regis A, St. Catherine E. Effects of an Entertainment-Education Radio Soap Opera on Family Planning and HIV Prevention in St. Lucia. *International Family Planning Perspectives* 2000;26(4):148-57. doi: 10.2307/2648250
33. Black A, Car J, Majeed A, et al. Strategic considerations for improving the quality of eHealth research: we need to improve the quality and capacity of academia to undertake informatics research. *Inform Prim Care* 2008;16(3):175-7. doi: 10.14236/jhi.v16i3.690 [published Online First: 2008/12/20]
34. Free C, Phillips G, Galli L, et al. The Effectiveness of Mobile-Health Technology-Based Health Behaviour Change or Disease Management Interventions for Health Care Consumers: A Systematic Review. *PLOS Medicine* 2013;10(1):e1001362. doi: 10.1371/journal.pmed.1001362
35. Free C, Phillips G, Watson L, et al. The Effectiveness of Mobile-Health Technologies to Improve Health Care Service Delivery Processes: A Systematic Review and Meta-Analysis. *PLOS Medicine* 2013;10(1):e1001363. doi: 10.1371/journal.pmed.1001363
36. NRLM. SHGs Profile Entry Status: Ministry of Rural Development, Government of India; 2020 [Available from: https://nrlm.gov.in/shgReport.do?methodName=showIntensiveStateWiseReport accessed 13 september 2020.
37. Singh R, Neogi SB, Hazra A, et al. Utilization of maternal health services and its determinants: a cross-sectional study among women in rural Uttar Pradesh, India. *Journal of Health, Population and Nutrition* 2019;38(1):13. doi: 10.1186/s41043-019-0173-5
38. Azad K, Barnett S, Banerjee B, et al. Effect of scaling up women's groups on birth outcomes in three rural districts in Bangladesh: a cluster-randomised controlled trial. *The Lancet* 2010;375(9721):1193-202. doi: https://doi.org/10.1016/S0140-6736(10)60142-0
39. Manandhar DS, Osrin D, Shrestha BP, et al. Effect of a participatory intervention with women's groups on birth outcomes in Nepal: cluster-randomised controlled trial. *The Lancet* 2004;364(9438):970-79. doi: https://doi.org/10.1016/S0140-6736(04)17021-9
40. Prost A, Colbourn T, Seward N, et al. Women's groups practising participatory learning and action to improve maternal and newborn health in low-resource settings: a systematic review and meta-analysis. *The Lancet* 2013;381(9879):1736-46. doi: https://doi.org/10.1016/S0140-6736(13)60685-6
41. Saggurti N, Atmavilas Y, Porwal A, et al. Effect of health intervention integration within women's self-help groups on collectivization and healthy practices around reproductive, maternal, neonatal and child health in rural India. *PLoS One* 2018;13(8):e0202562. doi: 10.1371/journal.pone.0202562
42. Saggurti N, Porwal A, Atmavilas Y, et al. Effect of behavioral change intervention around new-born care practices among most marginalized women in self-help groups in rural India: analyses of three cross-sectional surveys between 2013 and 2016. *J Perinatol* 2019;39(7):990-99. doi: 10.1038/s41372-019-0358-1 [published Online First: 2019/04/02]
43. Saha S, Kermode M, Annear PL. Effect of combining a health program with a microfinance-based self-help group on health behaviors and outcomes. *Public Health* 2015;129(11):1510-18. doi: https://doi.org/10.1016/j.puhe.2015.07.010
44. Seward N, Neuman M, Colbourn T, et al. Effects of women’s groups practising participatory learning and action on preventive and care-seeking behaviours to reduce neonatal mortality: A meta-analysis of cluster-randomised trials. *PLOS Medicine* 2017;14(12):e1002467. doi: 10.1371/journal.pmed.1002467
45. Tripathy P, Nair N, Barnett S, et al. Effect of a participatory intervention with women's groups on birth outcomes and maternal depression in Jharkhand and Orissa, India: a cluster-randomised

For peer review only - http://bmjopen.bmj.com/site/about/guidelines.xhtml

controlled trial. *The Lancet* 2010;375(9721):1182-92. doi: https://doi.org/10.1016/S0140-6736(09)62042-0
46. Walia M, Irani L, Chaudhuri I, et al. Effect of sharing health messages on antenatal care behavior among women involved in microfinance-based self-help groups in Bihar India. *Global Health Research and Policy* 2020;5(1):3. doi: 10.1186/s41256-020-0132-0
47. World Health Organization. Indicators for assessing infant and young child feeding practices: part II: measurement. Geneva, 2010:81.
48. Chakraborty D, Ahmad MS, Seth A. Findings from a Civil Society Mediated and Technology Assisted Grievance Redressal Model in Rural India. Proceedings of the Ninth International Conference on Information and Communication Technologies and Development. Lahore, Pakistan: Association for Computing Machinery, 2017:Article 2.
49. Moitra A, Das V, Vaani G, et al. Design Lessons from Creating a Mobile-based Community Media Platform in Rural India. Proceedings of the Eighth International Conference on Information and Communication Technologies and Development. Ann Arbor, MI, USA: Association for Computing Machinery, 2016:Article 14.
50. Grant A, Treweek S, Dreischulte T, et al. Process evaluations for cluster-randomised trials of complex interventions: a proposed framework for design and reporting. *Trials* 2013;14(1):15. doi: 10.1186/1745-6215-14-15
51. Moore G, Audrey S, Barker M, et al. Process evaluation in complex public health intervention studies: the need for guidance. *J Epidemiol Community Health* 2014;68(2):101-02. doi: 10.1136/jech-2013-202869 [published Online First: 2013/09/10]
52. Oakley A, Strange V, Bonell C, et al. Process evaluation in randomised controlled trials of complex interventions. *Bmj* 2006;332(7538):413-6. doi: 10.1136/bmj.332.7538.413 [published Online First: 2006/02/18]
53. Stead WW, Lin HS. Computational Technology for Effective Health Care: Immediate Steps and Strategic Directions. Washington (DC): National Academies Press (US) 2009.
54. White H. Theory-based impact evaluation: principles and practice. *Journal of Development Effectiveness* 2009;1(3):271-84. doi: 10.1080/19439340903114628
55. IIPS. Bihar state fact sheet: National Family Health Survey (NFHS-4), 2015–16. International Institute for Population Sciences (IIPS). Mumbai, India, 2017.
56. IIPS, ICF. National Family Health Survey (NFHS-4), 2015–16. International Institute for Population Sciences (IIPS). Mumbai, India, 2017.
57. TRAI. The Indian Telecom Services Performance Indicators April – June, 2019. New Delhi, India: Telecom Regulatory Authority of India, 2019a:131.
58. TRAI. The Indian Telecom Services Performance Indicators October – December, 2018. New Delhi, India: Telecom Regulatory Authority of India, 2019b:130.
59. TRAI. The Indian Telecom Services Performance Indicators July – September, 2019. New Delhi, India: Telecom Regulatory Authority of India, 2020:134.
60. Chen H-T, Rossi PH. The theory-driven approach to validity. *Evaluation and Program Planning* 1987;10(1):95-103. doi: https://doi.org/10.1016/0149-7189(87)90025-5
61. Chen HT, Chen H-t. Practical program evaluation: Assessing and improving planning, implementation, and effectiveness: Sage 2005.
62. Cohen CR, Steinfeld RL, Weke E, et al. Shamba Maisha: Pilot agricultural intervention for food security and HIV health outcomes in Kenya: design, methods, baseline results and process evaluation of a cluster-randomized controlled trial. *SpringerPlus* 2015;4(1):122. doi: 10.1186/s40064-015-0886-x
63. Dalkin SM, Greenhalgh J, Jones D, et al. What's in a mechanism? Development of a key concept in realist evaluation. *Implementation Science* 2015;10(1):49. doi: 10.1186/s13012-015-0237-x

For peer review only - http://bmjopen.bmj.com/site/about/guidelines.xhtml

For peer review only

64. Vassilev I, Rogers A, Kennedy A, et al. The influence of social networks on self-management support: a metasynthesis. *BMC Public Health* 2014;14(1):719. doi: 10.1186/1471-2458-14-719
65. Weiss CH. Theory-based evaluation: Past, present, and future. *New Directions for Evaluation* 1997;1997(76):41-55. doi: 10.1002/ev.1086
66. Devadasan N, Elias MA, John D, et al. A conditional cash assistance programme for promoting institutional deliveries among the poor in India: process evaluation results. *Reducing financial barriers to obstetric care in low-income countries* 2008
67. Filmer D, Pritchett LH. Estimating Wealth Effects Without Expenditure Data—Or Tears: An Application To Educational Enrollments In States Of India*. *Demography* 2001;38(1):115-32. doi: 10.1353/dem.2001.0003
68. Rutstein SO, Johnson K. The DHS wealth index. DHS comparative reports no. 6. Calverton: ORC Macro, 2004.
69. Angrist JD, Pischke J-S. Mostly harmless econometrics: An empiricist's companion: Princeton university press 2008.
70. Angrist JD, Pischke J-S. Mastering'metrics: The path from cause to effect: Princeton University Press 2014.
71. Ravallion M. Evaluating Anti-Poverty Programs. World Bank Policy Research. Working Paper 3625. Development Research Group, 2005.
72. Kumar GA, Dandona R, Chaman P, et al. A population-based study of neonatal mortality and maternal care utilization in the Indian state of Bihar. *BMC Pregnancy and Childbirth* 2014;14(1):357. doi: 10.1186/1471-2393-14-357
73. World Health Organization. Essential newborn care : report of a technical working group (Trieste, 25-29 April 1994). Geneva: World Health Organization, 1996.
74. Bergström A, Fottrell E, Hopkins H, et al. mHealth: can mobile technology improve health in low-and middle-income countries. *UCL public policy briefing* 2015
75. Dahdah MA, Kumar A. Mobile Phones for Maternal Health in Rural Bihar. *Economic & Political Weekly* 2018;53(11):51.
76. Ames HM, Glenton C, Lewin S, et al. Clients' perceptions and experiences of targeted digital communication accessible via mobile devices for reproductive, maternal, newborn, child, and adolescent health: a qualitative evidence synthesis. *Cochrane Database Syst Rev* 2019;10(10):CD013447-CD47. doi: 10.1002/14651858.CD013447
77. Mohan D, Bashingwa JJH, Tiffin N, et al. Does having a mobile phone matter? Linking phone access among women to health in India: An exploratory analysis of the National Family Health Survey. *PLoS One* 2020;15(7):e0236078. doi: 10.1371/journal.pone.0236078

For peer review only - http://bmjopen.bmj.com/site/about/guidelines.xhtml

BMJ Open

Key learnings from an outcome and embedded process evaluation of a direct to beneficiary mobile health intervention among marginalized women in rural Bihar, India

Journal:	*BMJ Open*
Manuscript ID	bmjopen-2021-052336.R1
Article Type:	Original research
Date Submitted by the Author:	16-May-2022
Complete List of Authors:	Irani, Laili; Population Council<br>., Supriya; Population Council<br>Mathur, Ruchika; Population Council<br>Verma, Raj Kumar; Population Council<br>Mohan, Diwakar; Johns Hopkins University Bloomberg School of Public Health<br>Dhar, Diva; Bill & Melinda Gates Foundation<br>Seth, Aaditeshwar; Gram Vaani<br>Chaudhuri , Indrajit; Project Concern International<br>Chaudhury, Mahua; JEEViKA, Bihar Rural Livelihoods Promotion Society, Department of Rural Development, Government of Bihar<br>Purthy, Apolo; JEEViKA, Bihar Rural Livelihoods Promotion Society, Department of Rural Development, Government of Bihar<br>Nanda, Ankit; Population Council<br>Singh, Shivani; Population Council<br>Gupta, Akshay; Gram Vaani<br>LeFevre, Amnesty; University of Cape Town
**Primary Subject Heading**:	Global health
Secondary Subject Heading:	Health services research, Medical education and training, Nutrition and metabolism, Public health, Qualitative research
Keywords:	Nutrition < TROPICAL MEDICINE, Human resource management < HEALTH SERVICES ADMINISTRATION & MANAGEMENT, EDUCATION & TRAINING (see Medical Education & Training)

SCHOLARONE™
Manuscripts

I, the Submitting Author has the right to grant and does grant on behalf of all authors of the Work (as defined in the below author licence), an exclusive licence and/or a non-exclusive licence for contributions from authors who are: i) UK Crown employees; ii) where BMJ has agreed a CC-BY licence shall apply, and/or iii) in accordance with the terms applicable for US Federal Government officers or employees acting as part of their official duties; on a worldwide, perpetual, irrevocable, royalty-free basis to BMJ Publishing Group Ltd ("BMJ") its licensees and where the relevant Journal is co-owned by BMJ to the co-owners of the Journal, to publish the Work in this journal and any other BMJ products and to exploit all rights, as set out in our licence.

The Submitting Author accepts and understands that any supply made under these terms is made by BMJ to the Submitting Author unless you are acting as an employee on behalf of your employer or a postgraduate student of an affiliated institution which is paying any applicable article publishing charge ("APC") for Open Access articles. Where the Submitting Author wishes to make the Work available on an Open Access basis (and intends to pay the relevant APC), the terms of reuse of such Open Access shall be governed by a Creative Commons licence – details of these licences and which Creative Commons licence will apply to this Work are set out in our licence referred to above.

Other than as permitted in any relevant BMJ Author's Self Archiving Policies, I confirm this Work has not been accepted for publication elsewhere, is not being considered for publication elsewhere and does not duplicate material already published. I confirm all authors consent to publication of this Work and authorise the granting of this licence.

For peer review only

Key learnings from an outcome and embedded process evaluation of a direct to beneficiary mobile health intervention among marginalized women in rural Bihar, India

Laili Irani, Supriya, Ruchika Tara Mathur, Raj Kumar Verma, Diwakar Mohan, Diva Dhar, Aaditeshwar Seth, Indrajit Chaudhuri, Mahua Roy Chaudhury, Apolo Purthy, Ankit Nanda, Shivani Singh, Akshay Gupta, Amnesty Lefevre

***Primary and corresponding author:**
Laili Irani
Postal address: c/o Population Council, Zone 5A, Ground Floor India Habitat Centre,
Lodi Road, New Delhi, Delhi 110003, India
Email: laili.ir@gmail.com; Telephone number: +91-1124642901, Fax number: +91-1124642903
ORCID iD: https://orcid.org/0000-0002-5472-507X

Co-authors:
Supriya: Population Council, New Delhi, India
Ruchika Tara Mathur: Population Council, New Delhi, India
Raj Kumar Verma: Population Council, New Delhi, India
Diwakar Mohan: Johns Hopkins University, Baltimore, U.S.A.
Diva Dhar: Bill and Melinda Gates Foundation, Seattle, U.S.A.
Aaditeshwar Seth: Gram Vaani, New Delhi, India
Indrajit Chaudhuri: Project Concern International, New Delhi, India
Mahua Roy Chaudhury: JEEViKA, Bihar Rural Livelihoods Promotion Society, Department of Rural Development, Government of Bihar, Patna, India
Apolo Purthy: JEEViKA, Bihar Rural Livelihoods Promotion Society, Department of Rural Development, Government of Bihar, Patna, India
Ankit Nanda: Population Council, New Delhi, India
Shivani Singh: Population Council, New Delhi, India
Akshay Gupta: Gram Vaani, New Delhi, India
Amnesty Lefevre: University of Cape Town Faculty of Science, Cape Town, South Africa

Keywords: mobile phone technology, interactive voice response system, self-help groups, Bihar, behavior change communication

Word count:
Abstract: 299 words
Manuscript: 5,501 words
Tables: 5
Figures: 3
References: 87

For peer review only - http://bmjopen.bmj.com/site/about/guidelines.xhtml

For peer review only

ABSTRACT

Introduction

Mobile Vaani was implemented as a pilot program across six blocks of Nalanda district in Bihar state, India to increase knowledge of rural women who were members of self-help groups on proper nutrition for pregnant or lactating mothers and infants, family planning, and diarrhea management. Conveners of self-help group meetings, community mobilizers, introduced women to the intervention by giving them access to interactive voice response informational and motivational content. A mixed methods outcome and embedded process evaluation was commissioned to assess the reach and impact of Mobile Vaani.

Methods

The outcome evaluation, conducted from January 2017 to November 2018, used a quasi-experimental pre-post design with a sample of 4,800 married women aged 15-49 from self-help group households, who had a livebirth in the past 24 months. Surveys with community mobilizers followed by meeting observations (n=116), in-depth interviews (n=180) with self-help group members, and secondary analyses of system generated data were conducted to assess exposure and perceptions of the intervention.

Results

From the outcome evaluation, 23% of women interviewed had heard about Mobile Vaani. Women in the intervention arm had significantly higher knowledge than women in the comparison arm for two of seven focus outcomes: knowledge of how to make child's food nutrient and energy dense (treatment-on-treated:18.8% (95% CI: 0.4-37.2, $p<0.045$)) and awareness of at least two modern spacing family planning methods (treatment-on-treated: 17.6% (95% CI: 4.7-30.5, $p<0.008$)). Women with any awareness of Mobile Vaani were happy with the program and appreciated the ability to call in and listen to the content.

Conclusion

For peer review only - http://bmjopen.bmj.com/site/about/guidelines.xhtml

Low population awareness and program exposure is underpinned by broader population level barriers to mobile phone access and use among women and missed opportunities by the program to improve targeting and program promotion. Further research is needed to assess programmatic linkages with changes in health practices.

For peer review only

Strengths and limitations of this study

- Strength: The evaluation utilized advanced analytical approaches to address low exposure in the outcome evaluation.
- Strength: The embedded process evaluation captured the design of how the program was integrated among self-help group members within a complex sociocultural environment.
- Limitation: Since, the measurement of exposure was based on reported estimates, future programs and evaluations should ensure that interactive voice recording call data records are accessible and linkable to survey data.

For peer review only

For peer review only - http://bmjopen.bmj.com/site/about/guidelines.xhtml

INTRODUCTION

When the National Family and Health Survey-4 results came out in 2016-2017, Bihar, India's third largest state (population 99 million), had lower than national averages for most maternal and child health outcomes.[1] In rural Bihar, half the children under five and nearly a third (32%) of mothers were underweight as compared to the national figures of 38% and 27%, respectively.[1 2] One in five (21%) married women reported having an unmet need for family planning – an estimate twice that of the national average. The incidence of diarrhea among children under-5 within the two weeks preceding the survey was 11% and less than half of those cases were managed with oral rehydration solution.[2] Against this backdrop, the government of Bihar, with the support of the Bill and Melinda Gates Foundation, established a range of public health programs to improve health systems and bolster health outcomes.

A maternal and child health and nutrition program was established in 2017, with implementation support provided by Project Concern International.[3] This program was designed to share health information with women who were members of microfinance-based self-help groups[1]. The target audience were mothers of children under two years of age who were either self-help group members themselves or were living in a home where at least one other woman residing in the same household was a member of a group. Prior studies had shown that sharing messages on correct maternal and child health and nutrition practices in weekly self-help group meetings coupled with home visits by group leaders and convenors of group meetings, called community mobilizers[2] to women with young children, among others, may improve some maternal and child health outcomes, such as antenatal care visits, early initiation of breastfeeding and clean cord care.[4-13] In an effort to bolster these and other outcomes, including family planning and complementary feeding, the government of Bihar sought to implement and evaluate new and innovative approaches including mobile health solutions which had experienced some success in reinforcing information to target audiences, within selected geographies.[4-8 14-49] Further,

[1] A self-help group comprises 8-12 women who live nearby, meet weekly, save small amounts of money to take out loans internally or with banks at small interest rates, and discuss matters related to finance, livelihoods and health.[3 8]
[2] Community mobilizers are women who support the functioning of ~10 self-help groups within their village. They facilitate weekly meetings for each self-help group.

For peer review only - http://bmjopen.bmj.com/site/about/guidelines.xhtml

as half the state's rural population owned a mobile phone, this new innovative program held potential.[30 50-52]

Mobile Vaani, developed by Gram Vaani and launched in January 2017, was a direct to beneficiary mobile health communication program comprised of a two-way messaging system which included outbound interactive voice response calls and a mechanism for beneficiaries to call in and record their own content. Trained community mobilizers, with support from program field officers, promoted awareness of the Mobile Vaani program in self-help group meetings. At regular intervals, group members were reminded about the program, and taught how to dial a toll-free number in order to generate a missed call. Within 30 seconds to two minutes of receiving the missed call, the system would call the number back and if answered, the listener received a series of pre-recorded World Health Organization-approved health information content on nutrition[53], family planning and management of diarrhea. Call content spanned across four priority health areas (child nutrition, maternal nutrition, family planning and management of diarrhea) and was comprised of two types of messages: those pre-recorded in a studio with actors reading off a script, or messages spontaneously recorded by self-help group women in the same district. The latter were reviewed and edited before being broadcast. Content was shared through story format which centered around the knowledge and benefits to women, children and families of appropriate maternal and child health and nutrition practices. Listeners were further encouraged to share information about Mobile Vaani with their family members, friends and neighbors, and to foster a dialogue and conversation with others on the topics listened to.

For peer review only

This manuscript presents findings from a mixed methods evaluation conducted with the support of JEEViKA, the government-led entity that establishes and runs self-help groups in Bihar. The evaluation was commissioned as very few interactive voice recording-based programs had been carried out targeting hard-to-reach marginalized populations and most of these programs had not been evaluated.[16 22 23] Further, even though Mobile Vaani had been implemented in the general population before, little was known about its reach, the perceptions of the content by its intended audience, or its impact on

For peer review only - http://bmjopen.bmj.com/site/about/guidelines.xhtml

knowledge for key maternal and child health and nutrition practices among women within self-help group households.[54 55]

Findings on the program's reach and impact on women's knowledge of key maternal and child health and nutrition indicators captured through the outcome evaluation are shared in this manuscript. It further highlights key results from an embedded process evaluation that helps to explain how the intervention was integrated into the existing system, how the intended beneficiaries engaged with the program and benefited from it, and how beneficiaries engaged with the technology. Study findings are anticipated to provide important insights into the impact direct to beneficiary solutions have in bolstering knowledge among women.

METHODS

Evaluation design and conceptual framework

The mixed methods evaluation design consisted of an outcome evaluation and an embedded process evaluation.

The outcome evaluation used a quasi-experimental before and after study design to identify changes in knowledge on maternal and child nutrition, family planning, and management of diarrhea among self-help group members with a child under 2 years of age. Six intervention blocks were selected by the implementers for programming; six comparison blocks were matched by the evaluators based on the socio-demographic characteristics of female literacy, scheduled caste/schedule tribe and concentration of self-help groups by population.

The process evaluation sought to garner in-depth knowledge on the functioning of Mobile Vaani, to assist in interpreting outcome results, and inform potential replication.[27 56-65] Using a realist evaluation approach, the process evaluation helped in building an overall understanding of (1) the system's readiness to accept the intervention, i.e., the ability of the community mobilizers to understand Mobile

For peer review only - http://bmjopen.bmj.com/site/about/guidelines.xhtml

Vaani and share its key tenets with their members, (2) a behavioral response to the program, as determined by the self-help group members' receptivity to accept, use and disseminate Mobile Vaani with others; and (3) the technological context within which the intervention was being implemented, that acted as an enabler or barrier to the uptake and utilization of the program (Figure 1).[66 67]

Data collection and sampling

Outcome evaluation: cross-sectional surveys

For the outcome evaluation, cross-sectional surveys were carried out at two time points: (1) baseline survey (n=2,436) from October 2017-January 2018; and (2) endline survey (n=2,498) 12 months following implementation from November 2018-January 2019 (Table 1). Cross-sectional surveys were powered to detect at least 7% net change in knowledge on core themes among the target population after 12 months of intervention. Assuming 80% power, and a design effect of 1.5, a sample size of 1,200 self-help group households in each arm was sufficient to detect this change. Study respondents were eligible women, aged 15-49 years, currently married, having had a live birth in the last 24 months preceding the survey and were from self-help group households. At endline, women were cross-sectionally selected from the same self-help groups. Less than 1% of the data collected across the variables of interest were missing and they were missing completely at random.

In the surveys, the women were asked about their engagement with Mobile Vaani and the intervention's impact on their knowledge of: maternal and child nutrition, family planning and management of diarrhea. Data were collected by female enumerators (n=40) with prior experience in conducting similar surveys. Enumerators were grouped in teams of five, including one designated mentor per team, and a supervisor who accompanied them to households, supported logistics, and checked completed study questionnaires. All data were captured on mini-laptops using CS-Pro version 7.1 software. Quality control occurred at three levels: (1) supervisory oversight of individual data collectors; (2) 10% spot checks of interviews by a team mentor; and (3) outlier analyses of entered data by the core research team.

For peer review only - http://bmjopen.bmj.com/site/about/guidelines.xhtml

The process evaluation was achieved through a triangulation of three primary data sources that were collected between November 2017-November 2018 and were used to measure the system's readiness and the behavioral response to the intervention (Table 1). Secondary data were analyzed to better understand the technological context around the intervention.

Process evaluation: system readiness

System readiness was measured through surveys with community mobilizers and observing self-help group meetings where community mobilizers talked about Mobile Vaani with their members. Surveys with 116 community mobilizers were carried out among those community mobilizers who had attended at least two trainings with field officers. After obtaining written consent from the respondents, data were captured in Hindi using computer-assisted personal interview software on laptops by trained female enumerators. Direct observations of self-help group meetings were conducted with the same community mobilizers who had been interviewed previously. These observations helped document what and how community mobilizers shared information about Mobile Vaani with their members. Trained investigators filled in structured questionnaires. Verbal consent was sought from all participants.

Process evaluation: behavioral response

Behavioral response was captured through 180 qualitative interviews carried out among selected self-help group women who knew of Mobile Vaani. They were interviewed in Hindi by trained investigators. Data were captured through audio recordings after obtaining written consent from the respondents. The individual interviews lasted an average of 40 minutes. Audio files were transcribed and then translated to English. Transcripts were imported into a qualitative software program, Atlas.ti version 6.2, for coding and in-depth analysis.

Process evaluation: technological context

The technological context was captured through secondary analyses of system-generated interactive voice recording data, which included 2.6 million call records from 44,664 unique phone numbers. Call

For peer review only - http://bmjopen.bmj.com/site/about/guidelines.xhtml

records, take took place from February 2017-July 2018, were obtained through the implementors and were analyzed to study calling and listening patterns and to identify any potential technical hindrances or barriers users may have faced.

Variables (Table 1)

Outcome evaluation: cross-sectional surveys

For the outcome evaluation, seven key dependent variables were measured. These included women's knowledge on three indicators related to child nutrition, namely: that complementary feeding for an infant was to be initiated between 6-12 months, what the correct consistency of food for a child 6-12 months old was, and how a child's food could be made nutrient and energy dense. The indicator capturing knowledge around maternal nutrition included the elements of adequate nutrition for a pregnant or lactating woman. Two indicators on family planning captured the respondents' knowledge of at least two modern spacing methods of family planning, and knowledge that frontline workers could provide short-term family planning methods, e.g., condoms/pills to women in need. The seventh indicator captured the interviewees' knowledge of the symptoms of diarrhea and its management. There were two key dichotomous independent variables of interest; one was the study arm, i.e., intervention or comparison, and the other was the survey round, i.e., baseline and endline. Control variables were included as these indicators were known to influence findings around the key dependent variables in previous studies.[1 68 69] These indicators were: women's parity (captured as an average measure across all the women in the category), number of completed years of formal education (categories: no formal education, completed 1-5 years of schooling, 6-9 years of schooling, and 10 or more years), any exposure to mass media over the past year (yes/no), belonging to the Hindu religion/not, belonging to a scheduled caste/tribe or not, household wealth(measured in quintiles)[3], respondent is a self-help group member/not, average duration of membership (in months), living in a nuclear family structure/not and

[3] The household wealth index was developed through principal component analysis using information on 26 household assets measured for six categories, five housing characteristics and one on asset ownership, taken from a nationally representative tool. Using these data, wealth quintiles were developed based on equal proportions of the population into five categories, from the first quintile being the poorest to the fifth quintile being the richest.

For peer review only - http://bmjopen.bmj.com/site/about/guidelines.xhtml

if the respondent had any contact with a frontline worker during pregnancy and seven days postpartum.[1 68 69]

Process evaluation: system readiness

In order to determine whether the community mobilizers were sharing adequate information about Mobile Vaani in their groups, community mobilizers were assessed on their knowledge around four key topics Mobile Vaani intended to share with self-help group women. This was captured through 31 knowledge-related questions asked to each of the community mobilizer with a correct answer to each question garnering a point; the scores for each of the responses were summed up to form a continuous variable. Through observations of self-help group meetings, community mobilizers were also assessed on whether they could demonstrate to their self-help group members how to dial a number on a mobile phone, how to access Mobile Vaani and the key features of the program. Other variables of interest were the number of messages community mobilizers reported being trained on (maximum being four: child nutrition, maternal nutrition, family planning and management of diarrhea); and other characteristics of the community mobilizers, i.e., age (captured as an average), caste (belonging to a scheduled caste/tribe or not), education (completed 1-10 years of schooling, 11-12 years of schooling, and college/more), whether she is engaged in another occupation or not, number of years she has been a community mobilizers (0-2 years, 3-4 years, 5+ years), and exposure to none/partial access to a mobile phone or complete access to a mobile phone[4].

Process evaluation: behavioral response

From the qualitative interviews that were held with women, four broad themes were identified and are presented in this article as they shed light on the outcome evaluation and help explain some of the enablers and barriers to the uptake of the program. These themes include the women's perspectives on how Mobile Vaani was integrated into self-help group meetings, the content they preferred listening to, the stories they resonated with and shared with others, and some of the barriers they faced in

[4] Full exposure to mobile phone meant owning a phone or having access to one 24 hours a day, and knowing how to dial a number. Partial access meant one of the elements of full exposure were missing.

For peer review only - http://bmjopen.bmj.com/site/about/guidelines.xhtml

accessing the program. Key indicators such as the age and educational level of the women who were interviewed were also captured.

Process evaluation: technological context

The purpose for analyzing the system-generated interactive voice recording data was to capture the lifecycle of a phone number within the system. The key variables included: a missed call, i.e., the user called the program generating a missed call, but the call was never answered back; a call back where a missed call from the user generated a call back from the system within 30 minutes of the user calling the system; and, an outbound dial, where the system called a registered user at periodic intervals on its own accord. In addition, the calls sent by the system were further analyzed to determine whether they were answered or not answered by the users. If the calls were answered, the duration for which the users listened to the content were categorized in seconds and minutes. Further, reasons for why calls were not answered were categorized into network issues, problems generating from the program itself or from the user, such as, the user not picking up the call.

Statistical procedures

Outcome evaluation: cross-sectional surveys

Differences between women's individual characteristics, i.e., covariates, across the intervention arms between baseline and endline were tested using ordinary least-squares (for continuous variables) and through logistic regression models (for categorical variables) (Table 2). For instance, women's parity and self-help group membership duration were measured as continuous variables, and hence tested using ordinary least-squares regression. The remaining variables were categorical, and hence tested using logistic regression model. In addition, the findings were adjusted for random clustering effect at the block and village levels by using multilevel mixed-effects regression analyses.

To assess the net change in the seven outcome variables of interest from baseline to endline, two types of multivariate analyses were carried out: one, a difference-in-difference analysis and two, a treatment-on-the-treated approach was applied using instrumental variable analysis (Table 3). The instrument

For peer review only - http://bmjopen.bmj.com/site/about/guidelines.xhtml

For peer review only

variable was those who were exposed to Mobile Vaani —as captured by those women who recalled hearing about Mobile Vaani or who recalled listening to content on it— thus controlling for endogeneity to program exposure.[70-72] These analyses were adjusted for covariates and for random clustering effect at the block and village levels. Samples with missing data were dropped from the multivariate analyses as the sample lost was negligible. All analyses were done using Stata version 16.0 software program.

Process evaluation: system readiness

Bivariate and descriptive analyses were conducted on key indicators from the quantitative interviews conducted with community mobilizers and observations from self-help group meetings (Table 4). The aim of the analyses was to capture the association of three key indicators that assessed the capabilities of the community mobilizers (knowledge on Mobile Vaani themes, ability to dial a number and explain all the features of the program) with other characteristics of interest, for example, their level of training, their socio-demographic profile, etc.

Process evaluation: behavioral response

The interview guides that the investigators used in the field covered several topics that were of interest to the evaluation. Once the interviews had been transcribed and imported into the qualitative software program, the transcripts were tagged with 70 codes that were generated from coding the transcripts. The codes were then brought together under nine broad themes of which four relevant themes are shared here.

Process evaluation: technological context

To analyze system-generated data, large volumes of data on phone calls to and from the system were merged. Cascade analyses of unique mobile numbers was conducted to determine user's engagement with the Mobile Vaani platform over time. For this, a lifecycle of each phone number was created. While crafting out the journey of each unique number's lifecycle, all the calls made by or to Mobile Vaani by the number were considered as exposures to Mobile Vaani. Further, durations of each call and reasons for a call not going through were also studied.

For peer review only - http://bmjopen.bmj.com/site/about/guidelines.xhtml

Ethical approval

This evaluation was approved by the evaluation agency, Population Council's institutional review board. The public was not involved in the design, conduct, reporting, or dissemination plans of this research. Prior to an interview, participants received written information on the survey as well the assurance of confidentiality. Written consent was taken from all interviewees, and interviews were conducted in a private space.

For peer review only

RESULTS

Outcome evaluation

Table 2 present summary characteristics for the sample population used in the cross-sectional survey. Findings suggest that more than half the women had no formal education, >90% belonged to Hindu religion, and >30% were members of the most disadvantaged schedule caste/ tribe. While the majority of the sample population reported having had contact with a FLW during their last pregnancy, less than half had a postnatal visit with a FLW seven days post-delivery. Self-help group membership was generally high with about 61-78% of the respondents in each of the study arms being a member themselves; the remaining eligible women interviewed came from a household where another adult female family member was a self-help group member. On average, self-help group members had been associated with their group for almost three years. For most sociodemographic characteristics assessed, the differences between study arms were not statistically significant suggesting that randomization was successful for the study design.

At endline, 17% (n=223) of the women in the intervention arm could recall listening to Mobile Vaani content at least once. Among them, most recalled listening to content on the themes of maternal nutrition (38%), child nutrition (35%), family planning (13%) and diarrhea management (12%). Health and nutrition messages were reported by 77% of women listeners to be the most useful.

For peer review only - http://bmjopen.bmj.com/site/about/guidelines.xhtml

Table 3 depicts results from the difference-in-difference and treatment-on-treated effect of the Mobile Vaani intervention on knowledge for selected maternal and child health practices. Women's knowledge of the correct consistency of food for a child 6-12 months of age was significantly higher over time across study arms (difference-in-difference: 6.6% 1.1%-12.2%) p=0.019) but not based on awareness of Mobile Vaani (treatment-on-treated: 14.4% (-6.1%-35%), p=0.169). Women's awareness of the need to make a child's food nutrient and energy dense increased significantly across study arms (difference-in-difference: 9.3% (4.5%-14.1%) p=<0.001) and among those with any awareness of Mobile Vaani (treatment-on-treated: 18.8% (0.4%-37.2%) p=0.045). Women's knowledge of proper nutrition for pregnant or lactating women increased in both groups over time but more substantially in the comparison arm from baseline to endline (Intervention: 73.6% to 77.0%; Control: 69.5% to 80.9%; difference-in-difference: -8.0% (-12.7%- -3.3%) p=0.001). Significant differences were not observed among those with any awareness of Mobile Vaani as compared to those with no awareness (treatment-on-treated: -0.3% (-16.8%-16.2%) p=0.971). Women's awareness of at least two modern spacing methods of family planning was significantly higher in the comparison arm across study arms (difference-in-difference: -4.7 (-8.2% -1.2%), p=0.0009) but when exposure to Mobile Vaani was considered, the opposite trend was observed and, a 17.6% (4.7%-30.5%; p=0.008) increase in knowledge was observed amongst those with any awareness of Mobile Vaani. Any awareness of the symptoms of diarrhea in children under 2 years of age and its management was low overall but increased significantly across study arms (difference-in-difference: 6.0% (2.0%-9.9%) p=0.003). However, these findings did not stay significant when knowledge of symptoms and management of diarrhea was considered among those with awareness of Mobile Vaani.

Process evaluation: System readiness

Table 4 depicts the characteristics of community mobilizers who were responsible for leading self-help groups and sharing information with self-help group members on Mobile Vaani. Community mobilizers were an average of 34 years of age, with 56% having more than 10 years of formal schooling, and 13% coming from the disadvantaged scheduled caste/tribe groups. Community mobilizers had similar knowledge scores irrespective of background characteristics. However, those who had received two or

For peer review only - http://bmjopen.bmj.com/site/about/guidelines.xhtml

more trainings could recall more messages correctly. Community mobilizers who had worked for a greater duration of time (three/more years) in their role and those with higher education (more than 10 years of schooling) had greater competency in dialing a number and explaining Mobile Vaani to their members than their counterparts.

Process evaluation: Behavioral response

Findings from in-depth interviews on the integration of Mobile Vaani into self-help groups provided insights into the barriers and facilitators to Mobile Vaani use (Table 5). Community mobilizers were hailed as the key peer educators to integrate Mobile Vaani into self-help groups, by sharing various aspects of the intervention with members in self-help group meetings (Theme 1). There was a general affinity towards the content shared through the intervention and reasons cited for this included it being their only source of news, and a useful medium for information on maternal health, nutrition and sanitation, which they could access at any time free of cost (Theme 2).

When it came to preferences, while most women expressed that they enjoyed listening to stories of local women from neighboring communities, their perceptions on the pre-recorded stories and messages generated in the studio were mixed with some younger and more educated women enjoying the messages that were woven into hypothetical scenarios with older and less educated women finding the content, language and dialect hard to understand and the pace of the storyline too fast, in general (Theme 3). Despite this, most women who were interviewed shared either the Mobile Vaani number or the information heard on Mobile Vaani at least once with their family members, neighbors and relatives with a few recounting stories of using the information they had learned to help others in dire need.

Qualitative findings suggest that there were economic, gendered and comprehension-related barriers, which hampered the uptake of the intervention (Theme 4). The most common reasons women shared that they did not listen to Mobile Vaani were a lack of time and no apparent benefit. Some women said that they had insufficient balance in their phones to give a missed call and a few women expressed doubts about Mobile Vaani being a free service. Beneficiaries also noted that calls came without

For peer review only - http://bmjopen.bmj.com/site/about/guidelines.xhtml

warning at timings that were not always convenient or when women had access to mobile phones. Some respondents with migrant husbands found it problematic to be on a Mobile Vaani call as their spouses complained that their (women's) phones were busy when the husbands called. Women also expressed apprehension in using mobile phones for long durations in front of male members of the household.

Technological context: Barriers to use

Beneficiaries could access Mobile Vaani either by answering an outbound call sent by Mobile Vaani or by directly giving a missed call (inbound call) and then being called back by the system. A review of the call records of the 44,664 users suggests that over a few exposures, the proportion of users receiving call-backs decreased, while the proportion of users who received random outbound calls being generated by the system increased (Figure 2).

An analysis of 2.6 million call records showed that around 19% of the calls were missed calls generated by the beneficiaries and 81% of all calls were made by Mobile Vaani to the users (Figure 3). Of these 81%, the majority were outbound calls (62%) made randomly by the system while the remaining 38% were call-backs generated as a result of a beneficiary sending a missed call to the system. Users' response to outbound dialed calls and call-backs were explored further and found that even though the system made more outbound calls than call-backs, the proportion of users answering the calls was much higher for call-backs as compared to outbound dialed calls, suggesting that call-backs were more cost effective for Mobile Vaani. If calls were initiated by users, 50-60% of the calls were answered as compared to calls that were pushed by the system (20-25%). A quarter of outbound dialed calls were disconnected within 30 seconds of answering, and users spent longer listening to messages on call-backs. Majority of the calls, irrespective of how they were received, were disconnected because of issues at the users' end which included 'normal clearing/no answer/call rejected/user busy'. Additionally, the reasons for call disconnection at Mobile Vaani end were network congestion, incapacity to handle a large volume of calls by primary rate interface lines, and other technical problems within the Mobile Vaani system (Figure 3).

For peer review only - http://bmjopen.bmj.com/site/about/guidelines.xhtml

DISCUSSION

This study presents results from a mixed methods evaluation of the Mobile Vaani program in two districts of Bihar. From the outcome evaluation, 23% of women from households with a self-help group member reported having heard about Mobile Vaani and 17% reported listening to content within the 12 months preceding the endline survey. Among women exposed to Mobile Vaani, qualitative findings suggest that they reportedly liked the program, found it convenient to access, and enjoyed recording and sharing content with friends and neighbors. However, the program was met with several existing contextual barriers such as lack of mobile literacy, poor phone infrastructure, inability to use mobile phones, gender and cultural barriers, lack of time, among others.[73] The program's impact on women's knowledge was mixed and limited to child nutrition and modern contraceptive methods. Overall findings underscore the challenges of trying to reach poor and marginalized groups of women with limited access to and skills to use mobile phones with digital interventions.

Study findings contribute to an emerging body of evidence on direct-to-consumer mobile health interventions in India.[74 75] The mobile messaging service Kilkari which aims to provide new and expectant mothers and their families with health information messages across 13 states in India similarly had a significant impact on a few but not all health behaviors assessed, namely reversible modern contracept methods and child immunizations at 10 weeks.[76] The limited impact observed there and in our evaluation of Mobile Vaani suggests that a more targeted approach to content delivery and one which considers differentials in digital access and skills may deepen impact.

Changing behaviors within communities takes time, concerted effort and reiteration of information on correct health practices, which can be achieved through repeated messaging and voice reminders using mobile phone technology through self-help groups.[19 77 78] However, it is important to note that health interventions using mobile phone technology may not function effectively in isolation but instead could be incorporated as a tool for improving indicators within a holistic health systems approach. In addition, community mobilizers also serve as additional conduits to reaching women with key health and

For peer review only - http://bmjopen.bmj.com/site/about/guidelines.xhtml

nutrition messaging, besides frontline workers who often bear the burden of being the primary interlocutors on health issues.[79-81] However, care should be taken not to overburden the community mobilizers whose primary role is to support self-help groups.

Mobile Vaani's impact on knowledge was driven by program coverage and exposure which were assessed based on reported awareness of and engagement with the program. Future evaluations might consider linking system generated data, including call data records, with survey data assessing outcomes. Population level coverage of Mobile Vaani among pregnant women was 23% based on reported survey estimates. An effort to validate this measure using call data records yielded a similar estimate. While the Kilkari program enrolled targeted women using government tracking registries, their overall population level coverage was 21%highlighting the challenge in reaching key audiences through mobile phone numbers within the Indian context.[82 83]

Reported exposure to Mobile Vaani was based on self-reported estimates and not call data records because of challenges linking varied data sources. Exposure was impeded by the limited number of self-help group members with mobile phones and children under 2 years of age (target population). Broader systematic challenges in women's access to and use of mobile phones persist in Bihar and elsewhere throughout India.[80 84 85] Women in the study sample reported having limited access to mobile phones, restricted by work and domestic commitments, coupled with limited digital skills. Further, patriarchal gender norms were omnipresent and restricted their use of mobile phones.[85 86] Similar contextual factors have been highlighted in explaining low outreach of mass media as well, including newspapers, radio and television, among women in rural Bihar.[1 2 26]

The evaluation highlighted the possibility to scale up the intervention with several caveats in mind. *First,* a space would need to be identified where a wide community could be reached that promoted ownership and sustenance of the intended intervention. *Second*, evidence-based and systematic planning would be needed to design a robust program to achieve its desired take-up by reaching intended audiences through capacity building and extensive promotion of the program. Adequate

For peer review only - http://bmjopen.bmj.com/site/about/guidelines.xhtml

coverage of the program would be essential for success. *Third*, the content being played through the interactive voice recording system would need to focus on topics of interest and be relatable to the audience to sustain its programming. As the audience would expand to new populations, this would require additional formative research and continued monitoring to tweak the program and allow for mid-course corrections. *Fourth*, for optimal results, the program ought to coordinate its content development and dissemination with any parallel program efforts disseminating health and nutrition information to the intended audience. *Fifth*, the program would need to identify and account for various socio-demographic and economic differences with the intent of reaching different audiences equally and not exacerbating disparities of access across socio-economic characteristics. Other scholars have also captured this phenomenon of intersecting inequalities, especially in terms of gendered phone access gaps and financial constraints.[73 80 87 88] *Sixth*, a robust registration and follow-up system of registered phone numbers would be pivotal in ensuring that messages would reach their intended audience at the appropriate time.

Though intervention and comparison arms were matched successfully, with the treatment and control groups generally comparable for their basic characteristics, this evaluation did have some limitations. Baseline and endline samples were not always balanced across the intervention and comparison arms, but key covariates were controlled for and accounted in the analysis. The study used fresh cross-sections of respondents at both time points of survey; however, respondents at endline belonged to the same self-help groups that were included at baseline, and therefore were representative of similar geographic and socio-economic conditions. As the study only engaged self-help group members, it may not have considerable external validity outside of this population. The exposure among the intended audience was low, but this was adjusted for in the analyses using an instrumental variable of recalled exposure to the program. The recall bias among respondents was also accounted for through rigorously trained investigators and pilot testing the survey tools, which were formulated based on established modules from similar surveys.

Conclusion

For peer review only - http://bmjopen.bmj.com/site/about/guidelines.xhtml

When introduced to Mobile Vaani through self-help groups, women's knowledge of key health and nutrition outcomes improved for two out of seven key indicators. The study provides evidence that mobile-based interventions could evolve into a modality to reach marginalized populations through systematic planning when coupled with other interventions on the ground. It would further be advisable for programs to remain conscious of the context, and enablers or barriers driven by socio-economic and gendered realities of the target audience, before operationalizing capital intensive interventions.

For peer review only

For peer review only - http://bmjopen.bmj.com/site/about/guidelines.xhtml

TABLES

Table 1: Details of data collection points

Data source	Study population	Sample	Date of data collection	Key outcomes	Key explanatory variables	Control variables	Presentation of results
**Outcome evaluation**							
**Structured quantitative interviews**	Eligible women	Baseline: 2,436 women<br>Endline: 2,498 women	Baseline: October 2016 – January 2017<br>Endline: November 2018 – January 2019	Knowledge on:<br>• Complementary feeding (Yes/No)<br>• Correct consistency of food for a child 6-12 months old (Yes/No)<br>• How to make a child's food nutrient rich and dense (Yes/No)<br>• Elements of adequate nutrition for a pregnant or lactating woman (Yes/No)<br>• Two modern spacing family planning methods (Yes/No)<br>• FLW as a source of short-term FP methods (Yes/No)<br>• How to identify and manage diarrhea among children (Yes/No)	• Study arm (Intervention and comparison arm)<br>• Survey time (baseline and endline)	• Women's parity (average)<br>• Number of completed years of formal education (no formal education, 1-5 years, 6-9 years, ≥10 years)<br>• Any exposure to mass media (i.e. radio, television or newspaper) over the past year (Yes/No)<br>• Religion (Hindu, Others)<br>• Scheduled caste/tribe (Yes/No)<br>• Household wealth (five quintiles)<br>• Self-help group membership (Yes/No)<br>• Duration of membership (Range: 0 – 180 months)<br>• Living in a nuclear family (Yes/No),<br>• Contact with a frontline worker during pregnancy (Yes/No)<br>• Contact with a frontline worker within 7 days of delivery (Yes/No)	Tables 2 and 3
**Process evaluation**							
**Structured quantitative interviews**	Community mobilizers	116	November 2017 –	Knowledge score of community mobilizers	• Number of trainings received	• Age (mean age)<br>• Caste (SC/ST, others)	Table 4

For peer review only - http://bmjopen.bmj.com/site/about/guidelines.xhtml

Data source	Study population	Sample	Date of data collection	Key outcomes	Key explanatory variables	Control variables	Presentation of results
			November 2018	around Mobile Vaani themes (Range: 0-31)	by community mobilizers on health and nutrition themes (0-1 theme, 2 themes, 3-4 themes)<br>•	• Education (1-10th std, 11th-12th std, college/more)<br>• Engaged in other occupation (yes/no)<br>• Duration of working as community mobilizer (0-2 years, 3-4 years, 5 or more years)<br>• Exposure to mobile phone (no/partial exposure, full exposure, i.e. having a personal phone, having access to a phone at all times, and ability to dial a number)	
**Direct observations**	Self-help group meetings	116	November 2017 – November 2018	• community mobilizer's ability to dial a number (Yes/No)<br>• community mobilizer's ability to explain Mobile Vaani features to women in a self-help group meeting (Yes/No)			
**In-depth qualitative interviews**	Community mobilizers<br>self-help group members<br>Program staff	55<br>180<br>18	November 2017 – November 2018	• Integrating Mobile Vaani into self-help groups<br>• Content preferred by users<br>• Type of content recollected and shared by respondents<br>• Reported barriers to accessing Mobile Vaani			Table 5
**System generated interactive voice recording data**	Phone numbers	2.6 million calls<br>44,664 unique phone numbers/ users	February 2017 – July 2018	• Missed calls - user called the system but the system did not call back<br>• Call backs - system called the user following a missed call from the user<br>• outbound calls - system-generated random calls to registered numbers at periodic intervals	• Answered calls - user answered incoming call<br>• Unanswered calls - user did not answer incoming call<br>• Duration of listenership, in minutes<br>• Reasons for disconnecting the call - network issues, Mobile Vaani issues, user issues		Figures 2 and 3

For peer review only

Table 2: Socio-demographic characteristics of the women interviewed in the cross-sectional surveys of the outcome evaluation

Socio-demographic characteristics	Baseline			Endline		
	Intervention (A) (N=1,249)	Comparison (B) (N=1,187)	p-value A vs B	Intervention (C) (N=1,188)	Comparison (D) (N=1,310)	p-value A vs C
**Women's parity,**						
**Mean (S.D.)**	2.95 (1.68)	3.06 (1.74)	0.142	2.81 (1.58)	2.91 (1.65)	0.008
**Women's education, %**						
**No formal education**	56.0	57.5	0.535	47.0	49.4	<0.001
**1-5 years**	12.3	14.5	0.112	13.9	14.7	0.200
**6-9 years**	15.8	15.2	0.729	20.0	20.2	0.005
**>=10 years of schooling**	15.9	12.8	0.064	19.2	16.7	0.020
**Any mass media exposure, %**						
**No**	62.9	74.5	<0.001	53.4	63.0	<0.001
**Yes**	37.1	25.5	<0.001	46.6	37.0	<0.001
**Belongs to Hindu religion, %**	98.5	91.1	<0.001	99.2	90.8	0.136
**Households' wealth quintile, %**						
**First**	13.6	28.4	<0.001	15.4	24.7	0.259
**Second**	18.3	21.1	0.126	19.6	20.0	0.383
**Third**	20.3	19.0	0.468	20.5	19.4	0.870
**Fourth**	23.8	15.8	<0.001	23.0	17.3	0.669
**Fifth**	24.1	15.7	<0.001	21.5	18.6	0.122
**Scheduled caste/tribe, %**						
**No**	68.6	69.3	0.815	69.6	65.5	0.654
**Yes**	31.4	30.7	0.815	30.4	34.5	0.654
**Woman herself is a self-help group member, %**						
**No**	31.9	21.9	<0.001	39.3	33.5	<0.001
**Yes**	68.1	78.1	<0.001	60.7	66.5	<0.001
**Women's self-help group membership duration in months**						
**Mean (S.D.)**	34.8 (20.1)	26.9 (17.9)	<0.001	38.5 (22.6)	36.1 (20.5)	0.001
**Nuclear family structure, %**						
**No**	67.4	57.3	<0.001	71.6	65.0	0.007
**Yes**	32.6	42.7	<0.001	28.4	35.0	0.007
**Any contact with FLW during pregnancy, %**						
**No**	27.8	43.0	<0.001	35.7	37.9	<0.001
**Yes**	72.2	57.0	<0.001	64.3	62.1	<0.001
**Any contact with FLW within 7 days of delivery, %**						
**No**	58.1	54.8	0.243	55.8	48.2	0.282
**Yes**	41.9	45.2	0.243	44.2	51.8	0.282

Note: Differences in groups at baseline and endline were tested by using ordinary least-squares regression models (continuous variables) or logit regression models (categorical variables), adjusting for clustering effect at the block and village level; S.D.: standard deviation

For peer review only - http://bmjopen.bmj.com/site/about/guidelines.xhtml

For peer review only

Table 3: Results from treatment-on-the-treated effect of Mobile Vaani intervention on knowledge of selected maternal and newborn health practices for outcome evaluation, 2017-2019

Indicators	Intervention		Comparison					
	Baseline (N=1,249)	Endline (N=1,188)	Baseline (N=1,187)	Endline (N=1,310)	Difference-in-difference estimate	p-value	Treatment-on-treated estimate	p-value
Knowledge indicators, %								
**Need to initiate complementary feeding for an infant at 6-12 months**	82.5	79.6	83.5	84.4	-3.8 (-8.0, 0.4)	0.077	-12.1 (-25.2, 0.9)	0.069
**Correct consistency of food is for a child aged 6-12 months**	55.6	56.4	56.0	50.2	6.6 (1.1, 12.2)	0.019	14.4 (-6.1, 35)	0.169
**How to make a child's food nutrient and energy dense**	72.6	79.8	74.3	72.2	9.3 (4.5, 14.1)	<0.001	18.8 (0.4, 37.2)	0.045
**Elements of adequate nutrition for a pregnant or lactating woman**	73.6	77.0	69.5	81.0	-8.0 (-12.7, -3.3)	0.001	-0.3 (-16.8, 16.2)	0.971
**Can name at least two modern spacing methods of family planning**	90.3	89.4	84.8	88.6	-4.7 (-8.2, -1.2)	0.009	17.6 (4.7, 30.5)	0.008
**Aware that frontline worker carries condoms and pills**	8.1	14.9	9.2	12.8	3.2 (-0.3, 6.7)	0.077	2.8 (-9.3, 15)	0.647
**Knows symptoms of diarrhea and how to manage it**	12.1	17.9	16.5	16.2	6.0 (2.0, 9.9)	0.003	-15.2 (-30.9, 0.4)	0.056

Note: Estimates are adjusted for women's parity, education, religion, caste, family structure, wealth quintile, mass media exposure, respondent's membership in groups and duration of self-help group membership, FLW contacts during pregnancy and after delivery, accounting for clustering effect at block and village level.
Treatment-on-treated effect are estimated based on instrument variable regression analysis and adjusted for women's parity, education, religion, caste, family structure, wealth quintile, mass media exposure, respondent's membership in groups and duration of self-help group membership, FLW contacts during pregnancy and after delivery, accounting for clustering effect at block and village level.

For peer review only

Table 4: Findings showing association of characteristics of community mobilizers to sharing Mobile Vaani in self-help group meetings (N=116)

Community mobilizers' background characteristics	Community mobilizers observed giving Mobile Vaani training in self-help group meetings	Ability of community mobilizers to give training on proposed topics during self-help group meetings: Average knowledge score Range, as captured during an interview (0-31)	Dial a number, as noted in a training/ self-help group meeting	Explain Mobile Vaani features to self-help group members in a meeting
	N (%)	Mean (SD)	Proportion (%)	Proportion (%)
**Number of health and nutrition messages that community mobilizers received training on**				
**0 – 1 theme**	14 (12.1)	8.2 (1.11)	57.1	64.3
**2 themes**	80 (69.0)	8.6 (1.22)	41.3	63.8
**3 – 4 themes**	22 (19.0)	8.9 (0.87)	31.8	45.5
**Age (mean, SD)**	33.3 (8.45)	8.6 (1.16)	31.7 (8.18)	33.0 (7.95)
**Caste, %**				
**SC/ST**	15 (12.9)	8.5 (1.21)	46.7	66.7
**Others**	101 (87.1)	8.6 (1.15)	40.6	59.4
**Education, %**				
**1st – 10th standard**	51 (44.0)	8.5 (1.05)	29.4	54.9
**11th – 12th standard**	39 (33.6)	8.7 (1.29)	46.2	64.1
**College and above**	26 (22.4)	8.7 (1.15)	57.7	65.4
**Engaged in other occupation, %**				
**Yes**	36 (31.0)	8.5 (1.03)	50.0	69.4
**No**	80 (69.0)	8.6 (1.19)	37.5	56.3
**Duration of work as a community mobilizer (years), %**				
**0-2 years**	32 (27.6)	8.5 (1.23)	28.1	31.3
**3-4 years**	48 (41.4)	8.8 (0.96)	43.8	68.8
**5+ years**	36 (31.0)	8.5 (1.27)	50.0	75.0
**Exposure to mobile phone, %**				
**No/partial exposure**	8 (6.9)	8.7 (1.55)	37.5	50.0
**Full exposure**	108 (93.1)	8.6 (1.11)	41.7	61.1

For peer review only

Table 5: Quotes from in-depth interviews with 180 self-help group women

Thematic reference in paper	Characteristics of women quoted	Quote
**Theme 1: Integrating Mobile Vaani into self-help groups**	30 years old, 12 years of education	*Community mobilizer is a homemaker. So, she spoke to us in the local dialect. We were all able to understand how we are supposed to dial the number, including the illiterate women of the [self-help].group.*
**Theme 2: Content preferred by users**	38 years old, 10 years of education	*I mostly like the voice of different women from villages as I feel motivated by listening to their stories. I also feel I should record my own voice just like they do.*
	35 years old, 9 years of education	*I like the studio voice more. The village women speak in breaks and they don't speak very well. But the studio sister speaks well and explains things very nicely.*
**Theme 3: Type of content recollected and shared by respondents**	30 years old, 3 years of education	*I have also discussed [about what I hear on Mobile Vaani] with my mother-in-law and sisters-in-law. My younger sister-in-law has a small baby and she needs to feed her baby twice or thrice in a day. She should feed her baby properly so the baby will remain healthy.*
**Theme 4: Reported barriers to accessing Mobile Vaani**	35 years old, 8 years of education	*You know how husbands are! They will start a fight with us if we are always busy with the mobile. I use it only in his absence and don't even touch it when he is home.*

Note: These quotes are an illustrative sample from a universe of 180 in-depth interviews conducted with self-help group women who had heard content on Mobile Vaani and engaged with the program in meetings as well.

For peer review only

For peer review only - http://bmjopen.bmj.com/site/about/guidelines.xhtml

Contributors: LI served as the PI of the study designing the evaluation, overseeing the data collection, leading the analysis and interpretation of results. LI was the primary author of the paper, had full access to the data, controlled the decision to publish and accepts full responsibility for the conduct of this study, as the guarantor. S, RM, RKM, AN and SS led the data collection, contributed to data analysis and visualization, and wrote early drafts of the methods and results sections of the manuscript. DM contributed to data analysis, interpretation of results and critical revision of the manuscript. DD and AL contributed to the plan and design of the study, contributed to data analysis, interpretation of results and critical revision of the manuscript for important intellectual content. AS, IC, MRC, AP, AG contributed to designing the study, assisted the evaluators to collect primary data and access secondary data, and provided critical insight into the interpretation of the results and revisions of the manuscript. All authors have reviewed and approved the final version of the manuscript.

Funding: This study was supported by the Bill and Melinda Gates Foundation [grant number OPP1141832].

Competing interests: None declared.

Ethics approval: The study was approved and annually reviewed by the evaluation agency, Population Council's institutional review board to which the primary author belongs. The approval number is 764.

Patient and public involvement: Patients and/or the public were not involved in the design, or conduct, or reporting, or dissemination plans of this research.

Patient consent for publication: Not required.

Provenance and peer review: Not commissioned; externally peer reviewed.

Data availability statement: Data are available upon request from the corresponding author. Requests for sharing data must be made of specific grounds, either (1) with the aim of corroborating the study results in the interest of public health, or (2) with the intention to answer new research questions. Sufficient information needs to be provided to confirm that the request is made for one of the above-mentioned purposes, including a sound justification, a protocol on the research for which the data will be used and a plan for quality control checks, as applicable.

For peer review only

Figure 1: Conceptual framework outlining key components of process and outcome evaluation

Figure 2: Cascade of unique numbers engaging with Mobile Vaani over the course of the program

Figure 3: User's engagement with Mobile Vaani system

For peer review only

For peer review only - http://bmjopen.bmj.com/site/about/guidelines.xhtml

REFERENCES

1. International Institute for Population Sciences (IIPS) and ICF. National Family Health Survey (NFHS-4), 2015–16. International Institute for Population Sciences (IIPS). Mumbai, India, 2017.
2. IIPS. Bihar state fact sheet: National Family Health Survey (NFHS-4), 2015–16. International Institute for Population Sciences (IIPS). Mumbai, India, 2017.
3. Irani L, Schooley J, Supriya, et al. Layering of a health, nutrition and sanitation programme onto microfinance-oriented self-help groups in rural India: results from a process evaluation. *BMC Public Health* 2021;21(1):2131. doi: 10.1186/s12889-021-12049-0
4. Azad K, Barnett S, Banerjee B, et al. Effect of scaling up women's groups on birth outcomes in three rural districts in Bangladesh: a cluster-randomised controlled trial. *The Lancet* 2010;375(9721):1193-202. doi: https://doi.org/10.1016/S0140-6736(10)60142-0
5. Manandhar DS, Osrin D, Shrestha BP, et al. Effect of a participatory intervention with women's groups on birth outcomes in Nepal: cluster-randomised controlled trial. *The Lancet* 2004;364(9438):970-79. doi: https://doi.org/10.1016/S0140-6736(04)17021-9
6. More NS, Bapat U, Das S, et al. Community Mobilization in Mumbai Slums to Improve Perinatal Care and Outcomes: A Cluster Randomized Controlled Trial. *PLOS Medicine* 2012;9(7):e1001257. doi: 10.1371/journal.pmed.1001257
7. Prost A, Colbourn T, Seward N, et al. Women's groups practising participatory learning and action to improve maternal and newborn health in low-resource settings: a systematic review and meta-analysis. *The Lancet* 2013;381(9879):1736-46. doi: https://doi.org/10.1016/S0140-6736(13)60685-6
8. Saggurti N, Atmavilas Y, Porwal A, et al. Effect of health intervention integration within women's self-help groups on collectivization and healthy practices around reproductive, maternal, neonatal and child health in rural India. *PLOS ONE* 2018;13(8):e0202562. doi: 10.1371/journal.pone.0202562
9. Saggurti N, Porwal A, Atmavilas Y, et al. Effect of behavioral change intervention around new-born care practices among most marginalized women in self-help groups in rural India: analyses of three cross-sectional surveys between 2013 and 2016. *J Perinatol* 2019;39(7):990-99. doi: 10.1038/s41372-019-0358-1 [published Online First: 2019/04/02]
10. Saha S, Kermode M, Annear PL. Effect of combining a health program with a microfinance-based self-help group on health behaviors and outcomes. *Public Health* 2015;129(11):1510-18. doi: https://doi.org/10.1016/j.puhe.2015.07.010
11. Seward N, Neuman M, Colbourn T, et al. Effects of women’s groups practising participatory learning and action on preventive and care-seeking behaviours to reduce neonatal mortality: A meta-analysis of cluster-randomised trials. *PLOS Medicine* 2017;14(12):e1002467. doi: 10.1371/journal.pmed.1002467
12. Tripathy P, Nair N, Barnett S, et al. Effect of a participatory intervention with women's groups on birth outcomes and maternal depression in Jharkhand and Orissa, India: a cluster-randomised controlled trial. *The Lancet* 2010;375(9721):1182-92. doi: https://doi.org/10.1016/S0140-6736(09)62042-0
13. Walia M, Irani L, Chaudhuri I, et al. Effect of sharing health messages on antenatal care behavior among women involved in microfinance-based self-help groups in Bihar India. *Global Health Research and Policy* 2020;5(1):3. doi: 10.1186/s41256-020-0132-0
14. Agarwal S, Perry HB, Long L-A, et al. Evidence on feasibility and effective use of mHealth strategies by frontline health workers in developing countries: systematic review. *Tropical Medicine & International Health* 2015;20(8):1003-14. doi: 10.1111/tmi.12525
15. Avula R, Menon P, Saha KK, et al. A Program Impact Pathway Analysis Identifies Critical Steps in the Implementation and Utilization of a Behavior Change Communication Intervention Promoting Infant and Child Feeding Practices in Bangladesh. *The Journal of Nutrition* 2013;143(12):2029-37. doi: 10.3945/jn.113.179085
16. Black A, Car J, Majeed A, et al. Strategic considerations for improving the quality of eHealth research: we need to improve the quality and capacity of academia to undertake informatics

For peer review only - http://bmjopen.bmj.com/site/about/guidelines.xhtml

research. *Inform Prim Care* 2008;16(3):175-7. doi: 10.14236/jhi.v16i3.690 [published Online First: 2008/12/20]

17. Chambers DA, Glasgow RE, Stange KC. The dynamic sustainability framework: addressing the paradox of sustainment amid ongoing change. *Implementation Science* 2013;8(1):117. doi: 10.1186/1748-5908-8-117
18. Donabedian A. The Quality of Care: How Can It Be Assessed? *JAMA* 1988;260(12):1743-48. doi: 10.1001/jama.1988.03410120089033
19. Feroz A, Perveen S, Aftab W. Role of mHealth applications for improving antenatal and postnatal care in low and middle income countries: a systematic review. *BMC Health Services Research* 2017;17(1):704. doi: 10.1186/s12913-017-2664-7
20. Feroz A, Rizvi N, Sayani S, et al. Feasibility of mHealth intervention to improve uptake of antenatal and postnatal care services in peri-urban areas of Karachi: A qualitative exploratory study. *Journal of Hospital Management and Health Policy* 2017;4(1)
21. Florez-Arango JF, Iyengar MS, Dunn K, et al. Performance factors of mobile rich media job aids for community health workers. *Journal of the American Medical Informatics Association* 2011;18(2):131-37. doi: 10.1136/jamia.2010.010025
22. Free C, Phillips G, Galli L, et al. The Effectiveness of Mobile-Health Technology-Based Health Behaviour Change or Disease Management Interventions for Health Care Consumers: A Systematic Review. *PLOS Medicine* 2013;10(1):e1001362. doi: 10.1371/journal.pmed.1001362
23. Free C, Phillips G, Watson L, et al. The Effectiveness of Mobile-Health Technologies to Improve Health Care Service Delivery Processes: A Systematic Review and Meta-Analysis. *PLOS Medicine* 2013;10(1):e1001363. doi: 10.1371/journal.pmed.1001363
24. Gurman TA, Rubin SE, Roess AA. Effectiveness of mHealth Behavior Change Communication Interventions in Developing Countries: A Systematic Review of the Literature. *Journal of Health Communication* 2012;17(sup1):82-104. doi: 10.1080/10810730.2011.649160
25. Källander K, Tibenderana JK, Akpogheneta OJ, et al. Mobile health (mHealth) approaches and lessons for increased performance and retention of community health workers in low-and middle-income countries: a review. *Journal of medical Internet research* 2013;15(1):e17.
26. Khan M, Bhatnagar I, Hazra A, et al. Situation analysis of behavior change communication activities in Bihar: A step towards developing a comprehensive BCC strategy. New Delhi: Population Council, 2012.
27. Labrique AB, Vasudevan L, Kochi E, et al. mHealth innovations as health system strengthening tools: 12 common applications and a visual framework. *Global Health: Science and Practice* 2013;1(2):160. doi: 10.9745/GHSP-D-13-00031
28. LeFevre AE, Mohan D, Hutchful D, et al. Mobile Technology for Community Health in Ghana: what happens when technical functionality threatens the effectiveness of digital health programs? *BMC Medical Informatics and Decision Making* 2017;17(1):27. doi: 10.1186/s12911-017-0421-9
29. Mahanta LB, Choudhury M, Borthakur S, et al. Can communication media improve the KAP status of frontline health workers? A cross-sectional study on MCH indicators from Assam, India. *South East Asia Journal of Public Health* 2015;5(2):57-64.
30. Malhotra A, Sharma R, Srinivasan R, et al. Widening the arc of indigenous communication: Examining potential for use of ICT in strengthening social and behavior change communication efforts with marginalized communities in India. *The Electronic Journal of Information Systems in Developing Countries* 2018;84(4):e12032. doi: 10.1002/isd2.12032
31. Mangwi Ayiasi R, Kolsteren P, Batwala V, et al. Effect of Village Health Team Home Visits and Mobile Phone Consultations on Maternal and Newborn Care Practices in Masindi and Kiryandongo, Uganda: A Community-Intervention Trial. *PloS one* 2016;11(4):e0153051-e51. doi: 10.1371/journal.pone.0153051

For peer review only - http://bmjopen.bmj.com/site/about/guidelines.xhtml

32. Maslowsky J, Frost S, Hendrick CE, et al. Effects of postpartum mobile phone-based education on maternal and infant health in Ecuador. *Int J Gynaecol Obstet* 2016;134(1):93-98. doi: 10.1016/j.ijgo.2015.12.008 [published Online First: 2016/04/08]
33. Mildon AM. Use of Mobile Phone Technology to Support Improved Infant and Young Child Feeding Practices in Low-and Middle-income Countries: A Scoping Review. University of Toronto (Canada), 2016.
34. Mitchell M, Hedt-Gauthier BL, Msellemu D, et al. Using electronic technology to improve clinical care – results from a before-after cluster trial to evaluate assessment and classification of sick children according to Integrated Management of Childhood Illness (IMCI) protocol in Tanzania. *BMC Medical Informatics and Decision Making* 2013;13(1):95. doi: 10.1186/1472-6947-13-95
35. Mozumdar A, Khan ME, Mondal SK, et al. Increasing knowledge of home based maternal and newborn care using self-help groups: Evidence from rural Uttar Pradesh, India. *Sexual & Reproductive Healthcare* 2018;18:1-9. doi: https://doi.org/10.1016/j.srhc.2018.08.003
36. Mushamiri I, Luo C, Iiams-Hauser C, et al. Evaluation of the impact of a mobile health system on adherence to antenatal and postnatal care and prevention of mother-to-child transmission of HIV programs in Kenya. *BMC Public Health* 2015;15(1):102. doi: 10.1186/s12889-015-1358-5
37. Nair N, Darak S, Parsekar S, et al. Effectiveness of Behaviour Change Communication interventions in improving the delivery of health messages for ante-natal care in limited literacy settings: An evidence summary. London: EPPI-Centre: Social Science Research Unit, UCL Institute of Education, University College London, 2017.
38. NRLM. SHGs Profile Entry Status: Ministry of Rural Development, Government of India; 2020 [Available from: https://nrlm.gov.in/shgReport.do?methodName=showIntensiveStateWiseReport accessed 13 september 2020.
39. Pagliari C, Sloan D, Gregor P, et al. What is eHealth (4): a scoping exercise to map the field. *Journal of medical Internet research* 2005;7(1):e9-e9. doi: 10.2196/jmir.7.1.e9
40. Philbrick WC. MHealth and MNCH: State of the Evidence. Trends, Gaps, Stakeholder Needs, and Opportunities For Future Research on the Use of Mobile Technology to Improve Maternal, Newborn, and Child Health. Washington: UN Foundation., 2013.
41. Pillai P. LEADING 21ST CENTURY HEALTHCARE: APP-CENTRIC CARE: "MOBILE HEALTH": The impact of Smartphone applications on health-a futuristic perspective on leading in complex healthcare system. *International Journal of Management Research and Reviews* 2012;2(4):518.
42. Rahman A, Leppard M, Rashid S, et al. Community perceptions of behaviour change communication interventions of the maternal neonatal and child health programme in rural Bangladesh: an exploratory study. *BMC Health Services Research* 2016;16(1):389. doi: 10.1186/s12913-016-1632-y
43. Rogers EM, Vaughan PW, Swalehe RMA, et al. Effects of an Entertainment-education Radio Soap Opera on Family Planning Behavior in Tanzania. *Studies in Family Planning* 1999;30(3):193-211. doi: 10.1111/j.1728-4465.1999.00193.x
44. Rothstein JD, Jennings L, Moorthy A, et al. Qualitative Assessment of the Feasibility, Usability, and Acceptability of a Mobile Client Data App for Community-Based Maternal, Neonatal, and Child Care in Rural Ghana. *International Journal of Telemedicine and Applications* 2016;2016:2515420. doi: 10.1155/2016/2515420
45. Singh N, Labrique A, Mehra S, et al. Can an Integrated mHealth System Increase Utilization of Antenatal care services and Promote Safe Delivery? 142nd APHA Annual Meeting and Exposition. New Orleans, LA, 2014.
46. Singh R, Neogi SB, Hazra A, et al. Utilization of maternal health services and its determinants: a cross-sectional study among women in rural Uttar Pradesh, India. *Journal of Health, Population and Nutrition* 2019;38(1):13. doi: 10.1186/s41043-019-0173-5

For peer review only - http://bmjopen.bmj.com/site/about/guidelines.xhtml

47. Smith C, Ngo TD, Gold J, et al. Effect of a mobile phone-based intervention on post-abortion contraception: a randomized controlled trial in Cambodia. *Bull World Health Organ* 2015;93(12):842-50A. doi: 10.2471/BLT.15.160267 [published Online First: 2015/10/15]
48. Uddin MJ, Shamsuzzaman M, Horng L, et al. Use of mobile phones for improving vaccination coverage among children living in rural hard-to-reach areas and urban streets of Bangladesh. *Vaccine* 2016;34(2):276-83. doi: 10.1016/j.vaccine.2015.11.024 [published Online First: 2015/11/29]
49. Vaughan PW, Regis A, St. Catherine E. Effects of an Entertainment-Education Radio Soap Opera on Family Planning and HIV Prevention in St. Lucia. *International Family Planning Perspectives* 2000;26(4):148-57. doi: 10.2307/2648250
50. TRAI. The Indian Telecom Services Performance Indicators April – June, 2019. New Delhi, India: Telecom Regulatory Authority of India, 2019a:131.
51. TRAI. The Indian Telecom Services Performance Indicators October – December, 2018. New Delhi, India: Telecom Regulatory Authority of India, 2019b:130.
52. TRAI. The Indian Telecom Services Performance Indicators July – September, 2019. New Delhi, India: Telecom Regulatory Authority of India, 2020:134.
53. World Health Organization. Indicators for assessing infant and young child feeding practices: part II: measurement. Geneva, 2010:81.
54. Chakraborty D, Ahmad MS, Seth A. Findings from a Civil Society Mediated and Technology Assisted Grievance Redressal Model in Rural India. Proceedings of the Ninth International Conference on Information and Communication Technologies and Development. Lahore, Pakistan: Association for Computing Machinery, 2017:Article 2.
55. Moitra A, Das V, Vaani G, et al. Design Lessons from Creating a Mobile-based Community Media Platform in Rural India. Proceedings of the Eighth International Conference on Information and Communication Technologies and Development. Ann Arbor, MI, USA: Association for Computing Machinery, 2016:Article 14.
56. Chen HT, Chen H-t. Practical program evaluation: Assessing and improving planning, implementation, and effectiveness: Sage 2005.
57. Chen H-T, Rossi PH. The theory-driven approach to validity. *Evaluation and Program Planning* 1987;10(1):95-103. doi: https://doi.org/10.1016/0149-7189(87)90025-5
58. Cohen CR, Steinfeld RL, Weke E, et al. Shamba Maisha: Pilot agricultural intervention for food security and HIV health outcomes in Kenya: design, methods, baseline results and process evaluation of a cluster-randomized controlled trial. *SpringerPlus* 2015;4(1):122. doi: 10.1186/s40064-015-0886-x
59. Dalkin SM, Greenhalgh J, Jones D, et al. What's in a mechanism? Development of a key concept in realist evaluation. *Implementation Science* 2015;10(1):49. doi: 10.1186/s13012-015-0237-x
60. Grant A, Treweek S, Dreischulte T, et al. Process evaluations for cluster-randomised trials of complex interventions: a proposed framework for design and reporting. *Trials* 2013;14(1):15. doi: 10.1186/1745-6215-14-15
61. Oakley A, Strange V, Bonell C, et al. Process evaluation in randomised controlled trials of complex interventions. *Bmj* 2006;332(7538):413-6. doi: 10.1136/bmj.332.7538.413 [published Online First: 2006/02/18]
62. Stead WW, Lin HS. Computational Technology for Effective Health Care: Immediate Steps and Strategic Directions. Washington (DC): National Academies Press (US) 2009.
63. Vassilev I, Rogers A, Kennedy A, et al. The influence of social networks on self-management support: a metasynthesis. *BMC Public Health* 2014;14(1):719. doi: 10.1186/1471-2458-14-719
64. Weiss CH. Theory-based evaluation: Past, present, and future. *New Directions for Evaluation* 1997;1997(76):41-55. doi: 10.1002/ev.1086
65. White H. Theory-based impact evaluation: principles and practice. *Journal of Development Effectiveness* 2009;1(3):271-84. doi: 10.1080/19439340903114628

For peer review only - http://bmjopen.bmj.com/site/about/guidelines.xhtml

66. Devadasan N, Elias MA, John D, et al. A conditional cash assistance programme for promoting institutional deliveries among the poor in India: process evaluation results. *Reducing financial barriers to obstetric care in low-income countries* 2008
67. Moore G, Audrey S, Barker M, et al. Process evaluation in complex public health intervention studies: the need for guidance. *J Epidemiol Community Health* 2014;68(2):101-02. doi: 10.1136/jech-2013-202869 [published Online First: 2013/09/10]
68. Filmer D, Pritchett LH. Estimating Wealth Effects Without Expenditure Data—Or Tears: An Application To Educational Enrollments In States Of India*. *Demography* 2001;38(1):115-32. doi: 10.1353/dem.2001.0003
69. Rutstein SO, Johnson K. The DHS wealth index. DHS comparative reports no. 6. Calverton: ORC Macro, 2004.
70. Angrist JD, Pischke J-S. Mostly harmless econometrics: An empiricist's companion: Princeton university press 2008.
71. Angrist JD, Pischke J-S. Mastering'metrics: The path from cause to effect: Princeton University Press 2014.
72. Ravallion M. Evaluating Anti-Poverty Programs. World Bank Policy Research. Working Paper 3625. Development Research Group, 2005.
73. Ames HM, Glenton C, Lewin S, et al. Clients' perceptions and experiences of targeted digital communication accessible via mobile devices for reproductive, maternal, newborn, child, and adolescent health: a qualitative evidence synthesis. *Cochrane Database Syst Rev* 2019;10(10):CD013447-CD47. doi: 10.1002/14651858.CD013447
74. Chakraborty A, Mohan D, Scott K, et al. Does exposure to health information through mobile phones increase immunisation knowledge, completeness and timeliness in rural India? *BMJ Global Health* 2021;6(Suppl 5):e005489. doi: 10.1136/bmjgh-2021-005489
75. Mohan D, Scott K, Shah N, et al. Can health information through mobile phones close the divide in health behaviours among the marginalised? An equity analysis of Kilkari in Madhya Pradesh, India. *BMJ Global Health* 2021;6(Suppl 5):e005512. doi: 10.1136/bmjgh-2021-005512
76. LeFevre AE, N. Shah KS, S. Chamberlain, O. Ummer, J.J.H. Bashingwa, A. Chakraborty, A. Godfrey, P. Dutt, D. Mohan. Are stage-based, direct to beneficiary mobile communication programs effective in improving maternal newborn and child health outcomes in India? Results from an individually randomised controlled trial of a national programme. *BMJ Global Health* 2022
77. Kumar GA, Dandona R, Chaman P, et al. A population-based study of neonatal mortality and maternal care utilization in the Indian state of Bihar. *BMC Pregnancy and Childbirth* 2014;14(1):357. doi: 10.1186/1471-2393-14-357
78. World Health Organization. Essential newborn care : report of a technical working group (Trieste, 25-29 April 1994). Geneva: World Health Organization, 1996.
79. Carmichael SL, Mehta K, Srikantiah S, et al. Use of mobile technology by frontline health workers to promote reproductive, maternal, newborn and child health and nutrition: a cluster randomized controlled Trial in Bihar, India. *J Glob Health* 2019;9(2):0204249-49. doi: 10.7189/jogh.09.020424
80. Dahdah MA, Kumar A. Mobile Phones for Maternal Health in Rural Bihar. *Economic & Political Weekly* 2018;53(11):51.
81. Ward V, Abdalla S, Raheel H, et al. Implementing health communication tools at scale: mobile audio messaging and paper-based job aids for front-line workers providing community health education to mothers in Bihar, India. *BMJ Global Health* 2021;6(Suppl 5):e005538. doi: 10.1136/bmjgh-2021-005538
82. Mohan D, Bashingwa JJH, Scott K, et al. Optimising the reach of mobile health messaging programs in India: An analysis of system generated data for the Kilkari program across 13 states in India. *Submitted for Publication*
83. Scott K, Ummer O, Chamberlain S, et al. At the frontlines of digitisation: a qualitative study on the challenges and opportunities in maintaining accurate, complete and timely digital health

For peer review only - http://bmjopen.bmj.com/site/about/guidelines.xhtml

records in India's government health system. *BMJ Open* 2022;12(2):e051193. doi: 10.1136/bmjopen-2021-051193

84. Bergström A, Fottrell E, Hopkins H, et al. mHealth: can mobile technology improve health in low- and middle-income countries. *UCL public policy briefing* 2015
85. Scott K, Ummer O, Shinde A, et al. Another voice in the crowd: the challenge of changing family planning and child feeding practices through mHealth messaging in rural central India. *BMJ Global Health* 2021;6(Suppl 5):e005868. doi: 10.1136/bmjgh-2021-005868
86. Scott K, Shinde A, Ummer O, et al. Freedom within a cage: how patriarchal gender norms limit women's use of mobile phones in rural central India. *BMJ Global Health* 2021;6(Suppl 5):e005596. doi: 10.1136/bmjgh-2021-005596
87. Mohan D, Bashingwa JJH, Tiffin N, et al. Does having a mobile phone matter? Linking phone access among women to health in India: An exploratory analysis of the National Family Health Survey. *PLOS ONE* 2020;15(7):e0236078. doi: 10.1371/journal.pone.0236078
88. Singh, R., et al., Utilization of maternal health services and its determinants: a cross-sectional study among women in rural Uttar Pradesh, India. Journal of Health, Population and Nutrition, 2019. 38(1): p. 13.

For peer review only

For peer review only - http://bmjopen.bmj.com/site/about/guidelines.xhtml

Figure 1: Conceptual framework outlining key components of process and outcome evaluation

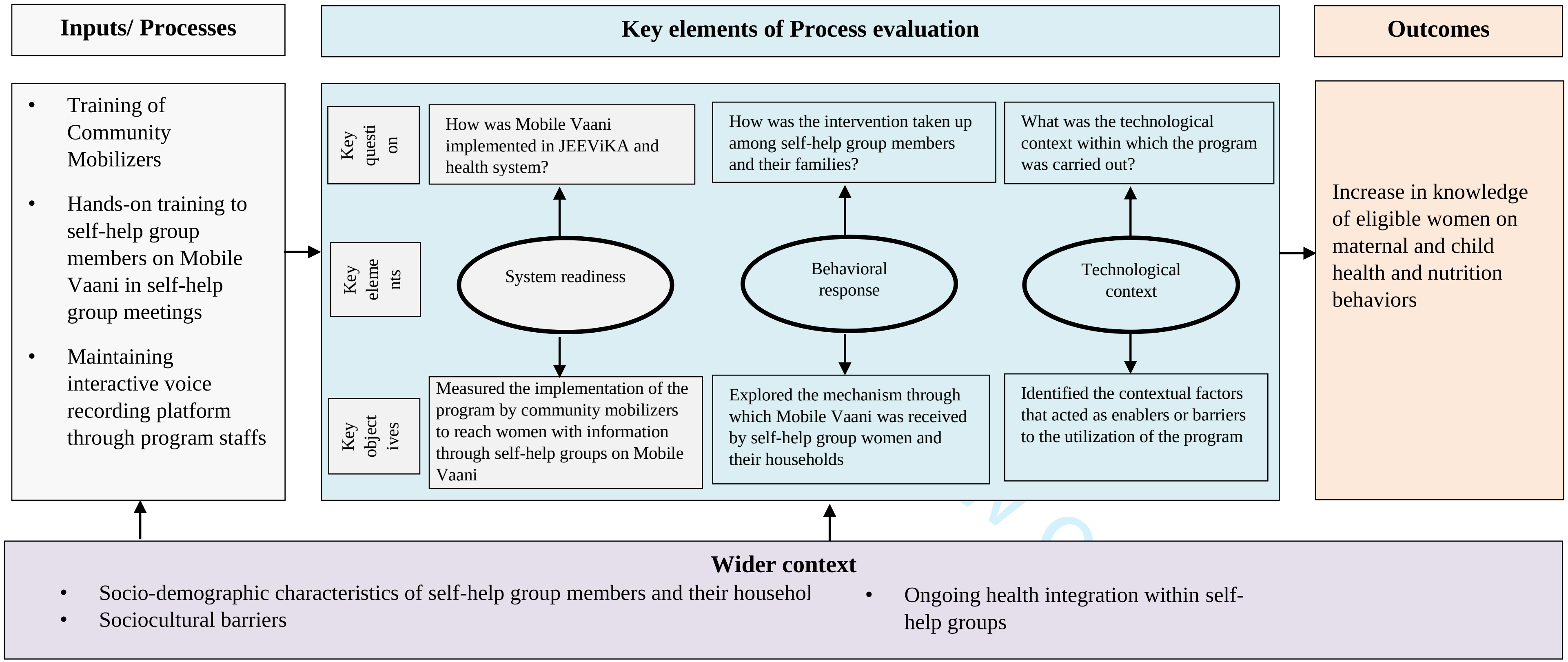

Figure 2: Cascade of unique numbers engaging with Mobile Vaani over the course of the program

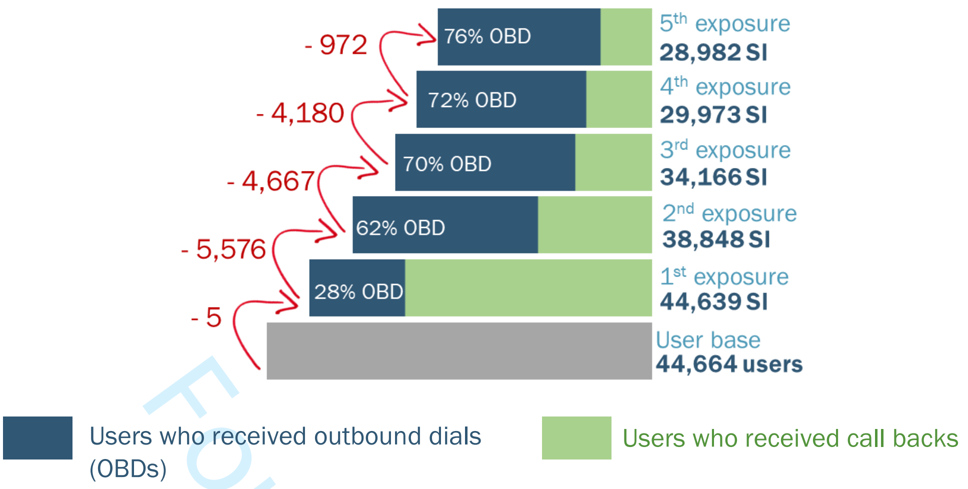

For peer review only

Figure 3: User's engagement with Mobile Vaani system

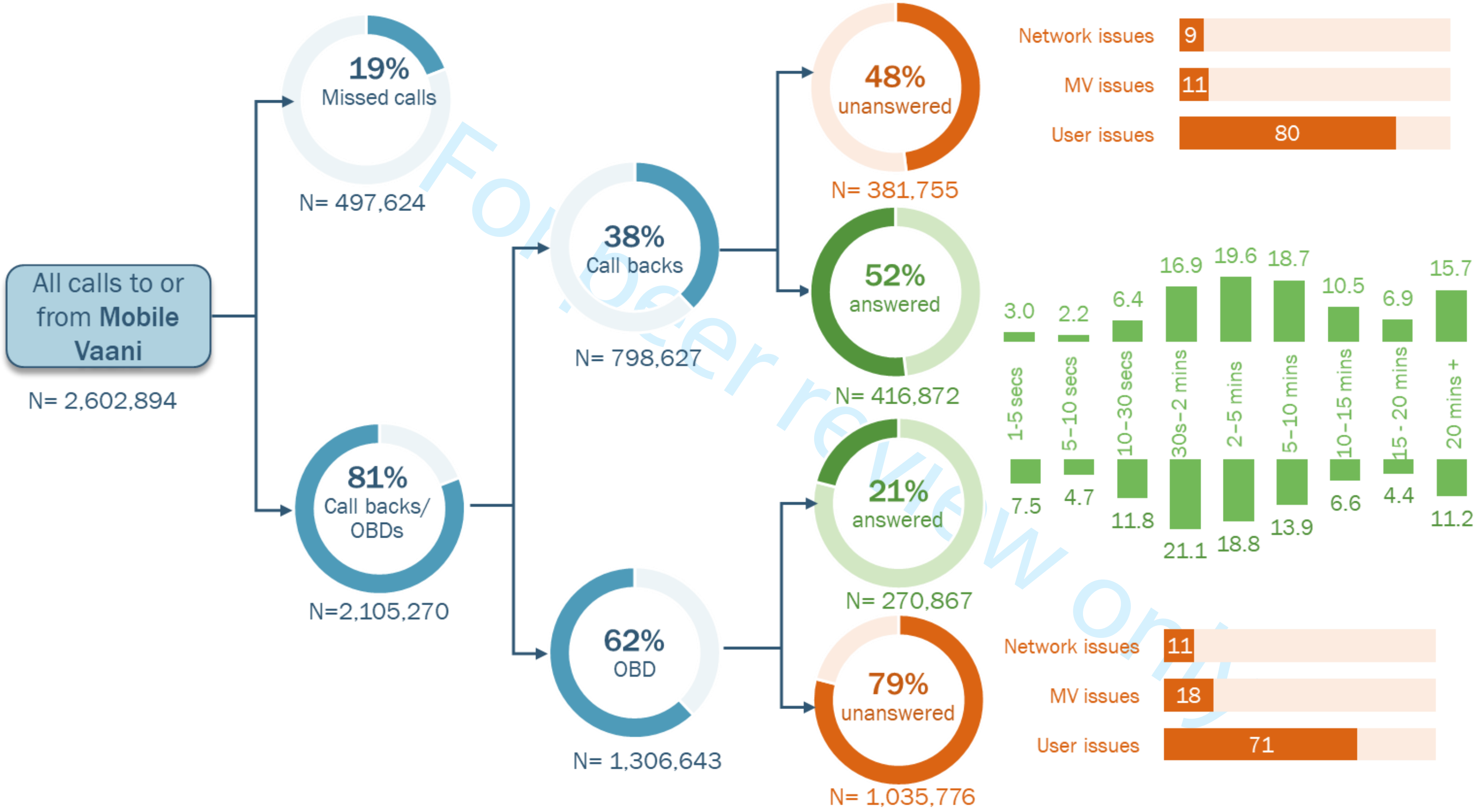

STROBE 2007 (v4) checklist of items to be included in reports of observational studies in epidemiology*
Checklist for cohort, case-control, and cross-sectional studies (combined)

Section/Topic	Item #	Recommendation	Reported on page #
**Title and abstract**	1	(*a*) Indicate the study's design with a commonly used term in the title or the abstract	1-3
		(*b*) Provide in the abstract an informative and balanced summary of what was done and what was found	2-3
**Introduction**			
Background/rationale	2	Explain the scientific background and rationale for the investigation being reported	5-6
Objectives	3	State specific objectives, including any pre-specified hypotheses	6-7
**Methods**			
Study design	4	Present key elements of study design early in the paper	7-8, 22
Setting	5	Describe the setting, locations, and relevant dates, including periods of recruitment, exposure, follow-up, and data collection	8-10
Participants	6	(*a*) *Cohort study*—Give the eligibility criteria, and the sources and methods of selection of participants. Describe methods of follow-up<br>*Case-control study*—Give the eligibility criteria, and the sources and methods of case ascertainment and control selection. Give the rationale for the choice of cases and controls<br>*Cross-sectional study*—Give the eligibility criteria, and the sources and methods of selection of participants	8-9
		(*b*) *Cohort study*—For matched studies, give matching criteria and number of exposed and unexposed<br>*Case-control study*—For matched studies, give matching criteria and the number of controls per case	Not applicable
Variables	7	Clearly define all outcomes, exposures, predictors, potential confounders, and effect modifiers. Give diagnostic criteria, if applicable	10-12
Data sources/ measurement	8*	For each variable of interest, give sources of data and details of methods of assessment (measurement). Describe comparability of assessment methods if there is more than one group	10-12
Bias	9	Describe any efforts to address potential sources of bias	8
Study size	10	Explain how the study size was arrived at	8
Quantitative variables	11	Explain how quantitative variables were handled in the analyses. If applicable, describe which groupings were chosen and why	10-11
Statistical methods	12	(*a*) Describe all statistical methods, including those used to control for confounding	12-13
		(*b*) Describe any methods used to examine subgroups and interactions	13
		(*c*) Explain how missing data were addressed	13
		(*d*) *Cohort study*—If applicable, explain how loss to follow-up was addressed<br>*Case-control study*—If applicable, explain how matching of cases and controls was addressed	12-13

For peer review only

		Cross-sectional study—If applicable, describe analytical methods taking account of sampling strategy	
		(*e*) Describe any sensitivity analyses	Not applicable
**Results**			
Participants	13*	(a) Report numbers of individuals at each stage of study—eg numbers potentially eligible, examined for eligibility, confirmed eligible, included in the study, completing follow-up, and analysed	14
		(b) Give reasons for non-participation at each stage	15-16
		(c) Consider use of a flow diagram	23-24
Descriptive data	14*	(a) Give characteristics of study participants (eg demographic, clinical, social) and information on exposures and potential confounders	14-17
		(b) Indicate number of participants with missing data for each variable of interest	8
		(c) *Cohort study*—Summarise follow-up time (eg, average and total amount)	Not applicable
Outcome data	15*	*Cohort study*—Report numbers of outcome events or summary measures over time	Not applicable
		Case-control study—Report numbers in each exposure category, or summary measures of exposure	Not applicable
		Cross-sectional study—Report numbers of outcome events or summary measures	14
Main results	16	(*a*) Give unadjusted estimates and, if applicable, confounder-adjusted estimates and their precision (eg, 95% confidence interval). Make clear which confounders were adjusted for and why they were included	15
		(*b*) Report category boundaries when continuous variables were categorized	10-11
		(*c*) If relevant, consider translating estimates of relative risk into absolute risk for a meaningful time period	Not applicable
Other analyses	17	Report other analyses done—eg analyses of subgroups and interactions, and sensitivity analyses	15-18
**Discussion**			
Key results	18	Summarise key results with reference to study objectives	18
Limitations	19	Discuss limitations of the study, taking into account sources of potential bias or imprecision. Discuss both direction and magnitude of any potential bias	20
Interpretation	20	Give a cautious overall interpretation of results considering objectives, limitations, multiplicity of analyses, results from similar studies, and other relevant evidence	18-21
Generalisability	21	Discuss the generalisability (external validity) of the study results	20
**Other information**			
Funding	22	Give the source of funding and the role of the funders for the present study and, if applicable, for the original study on which the present article is based	30

*Give information separately for cases and controls in case-control studies and, if applicable, for exposed and unexposed groups in cohort and cross-sectional studies.
Note: An Explanation and Elaboration article discusses each checklist item and gives methodological background and published examples of transparent reporting. The STROBE checklist is best used in conjunction with this article (freely available on the Web sites of PLoS Medicine at http://www.plosmedicine.org/, Annals of Internal Medicine at http://www.annals.org/, and Epidemiology at http://www.epidem.com/). Information on the STROBE Initiative is available at www.strobe-statement.org.

For peer review only
